# Comparison of long-term changes in size and longevity of bee colonies in mid-west Japan and Maui with and without exposure to pesticide, cold winters, and mites

**DOI:** 10.7717/peerj.9505

**Published:** 2020-07-28

**Authors:** Toshiro Yamada, Kazuko Yamada

**Affiliations:** 1 Graduate School of Natural Science & Technology, Kanazawa University, Kanazawa, Ishikawa, Japan; 2 Freelance, Kanazawa, Ishikawa, Japan

**Keywords:** Neonicotinoid, Organophosphate, Longevity, Dinotefuran, Fenitrothion, Honeybee, Clothianidin, Field experiment, Colony size, Maui

## Abstract

Four long-term field experiments in mid-west Japan (Shika) made it clear that extinction of colonies exposed to neonicotinoid was much higher than for colonies exposed to organophosphates. The incidence of hive death for of organophosphate-exposed and control (pesticide-free) colonies was similar. We conducted a field experiment in Maui for 271 days using the same pesticides (dinotefuran: 0.2 ppm, clothianidin: 0.08 ppm, fenitrothion: 1 ppm) as used in Shika with the honeybee, *Apis mellifera*, colonies without mites. Numbers of adult bees, capped brood, mites and other hive parameters were accurately counted on photographs of combs and on the inside of the hives. All six neonicotinoid (dinotefuran & clothianidin)-exposed colonies failed during the experiment. One of three organophosphate (fenitrothion)-exposed colonies and one of the three control colonies also failed. The findings from Maui, where colonies displayed no mites, provides evidence from Shika, with mites, that neonicotinoids are more hazardous to honeybee colonies than organophosphates. The apparent longevity of honeybee colonies on Maui was estimated by numbers of adult bees and capped brood using a mathematical model previously proposed. Seasonal changes in longevity on Maui differ greatly from changes at Shika, the latter showing distinct seasonal variation. Longevity on Maui remains nearly constant throughout the year with wide variations. At Shika, it increases drastically in winter, by six- to ten fold more than the other seasons. Differences seem to depend on the existence of cold winters and the length of flowering seasons. In a perpetually hospitable environment, small changes in conditions can be sensitively reflected in apparent longevity. Examining wide variations in apparent longevity that are seemingly incoherent, we recognized several differences in apparent longevity between neonicotinoid-exposed and organophosphate-exposed colonies: The colony that failed in after organophosphate-exposure colony group exhibited the longest apparent longevity and the fewest number of newly capped brood, as also was the case in control colonies. Extended longevity when few brood are newly produced is reasonable to maintain the colony from a physiological point of view. Extension of apparent longevity is not seen in neonicotinoid-exposed colonies when the number of newly capped brood is fewer. This finding suggests that neonicotinoid pesticides may inhibit normal apian physiology.

## Introduction

After a moratorium on the use of neonicotinoid pesticides in the European Union, many reports on their threats to ecosystems have been published (Tirado, Simon & Johnston, 2013; Van Lexmond, Bonmatin & Noome, 2015). We conducted four long-term field experiments in 2010 (Yamada, Yamada & Wada, 2012), 2011–2012 (Yamada, Yamada & Yamada, 2018a), 2012–2013 (Yamada, Yamada & Yamada, 2018b) and 2013–2014 (Yamada, Yamada & Yamada, 2018c) in mid-west of Japan (Shika) (Latitude 37°1′9″N, Longitude 165°46′14″E). This region has four distinct seasons and mites are present. We examined the impact of pesticides on the honeybee, *Apis mellifera*, colonies. Results made clear that neonicotinoids are much more likely to cause colony extinction than organophosphates. Colony collapse disorder seems a consequence of chronic toxicity. It was speculated that neonicotinoid pesticides inhibit egg-laying and decrease immune competence of bees leading to mite infestation (Yamada, Yamada & Wada, 2012). Di Prisco et al. (2013) demonstrated that exposure to the neonicotinoid, clothianidin, reduces immune defenses and promotes infection with deformed wing virus, vectored by the Varroa mite. This impact was not observed in honeybees exposed to the organophosphate, chlorpyriphos. Lu, Warchol & Callahan (2012, 2014) conducted similar field experiments in apiaries. A strong suspicion that neonicotinoid pesticides cause massive loss of bee colonies in the Northern Hemisphere is now changing to a conviction (Steinhauer et al., 2015; Brodschneider et al., 2016; Kulhanek et al., 2017). We reported neonicotinoids would be involved in massive colony loss, and that honeybee colonies weakened by these pesticides can be infested by mites. We seldom found mites until near the time of colony failure (Yamada, Yamada & Wada, 2012; Yamada, Yamada & Yamada, 2018a, 2018c). A few investigators, however, insisted that our study (Yamada, Yamada & Wada, 2012) was misinterpreted (Nakamura et al., 2014). To verify the validity of our findings in Shika (mid-west Japan), we conducted a long-term field experiment in Maui (Hawaii) where no mites harmful to honeybees exist (Ramadan et al., 2008; Santamaria et al., 2018), and no distinct seasonal changes occur. We used the same pesticides with the same concentrations as in Shika (mid-west Japan) where mites exist, and distinct seasonal changes occur (Yamada, Yamada & Apao, 2018).

The lifespan of honeybees is an important indicator of individual activity periods. Average and maximum lifespan in a honeybee colony is important as an indicator of the activity periods of the colony. It is almost impossible to directly measure seasonal changes in average or maximum lifespan in a honeybee colony composed of a few ten thousands of individual honeybees from their individual lifespans. Very few papers report on seasonal changes in longevity, defined as the age of the oldest group in a honeybee colony (Fukuda & Sekiguchi, 1966). Several mathematical models based on population dynamics are proposed to estimate seasonal lifespan changes (Biihlmann, 1985; Omholt, 1988; Tofilski, 2002, 2006; Khoury, Barron & Myerscough, 2013). These models incorporate several arbitrary parameters that provide abundant but possibly inaccurate and variable information. A higher number of arbitrary parameters will result in lower accuracy.

We proposed a mathematical model with no arbitrary parameters that provides an estimation of “apparent longevity”, meaning the age in days of longest surviving group in a honeybee colony as an indicator of its period (Yamada, Yamada & Yamada, 2019). Apparent longevity can be estimated using only the number of adult bees and capped brood. Control colonies into which pesticide-free sugar syrup was fed showed similar seasonal changes in apparent longevity to one another among three long-term field experiments conducted in Shika. Apparent longevity began to increase in the latter half of September, increased rapidly from the latter half of October with the approach of winter, reached a maximum in the latter half of April in the ensuing year, then decreased dramatically (Yamada, Yamada & Yamada, 2019).

The above findings come from studies in mid-west of Japan, where seasonal changes are clear-cut, but whether the above findings are applicable to locations where changes are not distinct, with no cold winter, such as in Hawaii is debatable. In this work, we compare seasonal changes in bee size and apparent longevity for control colonies where pesticide-free sugar syrup is fed and pesticide-exposed colonies where pesticide was fed using sugar syrup. Study sites were Maui (Hawaii) without clear seasons (especially no cold winter) and Shika (mid-west Japan) with clear seasons.

## Materials and Methods

### Ethics statement

No specific permissions were required for these locations/activities because the apiary at which we conducted experiments belongs to the collaborator Mr. Apao Paul (see “Acknowledgment”). We confirm that field studies did not involve endangered or protected species.

### Materials and preparation of pesticide concentrations

We used pesticides from Starkle Mate^®^ (10% dinotefuran; Mitsui Chemicals Aglo, Inc., Tokyo, Japan), Dantotsu^®^ (16% clothianidin; Sumitomo Co. Ltd., Tokyo, Japan) and Sumithion^®^ emulsion (50% fenitrothion; Sumitomo Co. Ltd., Tokyo, Japan). We dissolved pesticides in a sugar syrup made by mixing cane sugar (purchased from a Maui COSTCO wholesale supermarket) with an equal weight of water and added pesticides at the desired concentrations. Bees were fed pesticide containing sugar syrup and compare with colonies receiving syrup with no pesticides. Sugar syrups with pesticide were prepared to represent equal insecticidal activity (0.2 ppm for dinotefuran, 0.08 ppm for clothianidin, 1 ppm for fenitrothion). These concentration display approximately the same insecticidal activity for stink bugs. These concentrations are much lower than a concentration detected near rice paddies in Japan. A concentration of about 5 ppm of clothianidin near a rice field was reported by Kakuta et al. (2011) and about 0.7 ppm of dinotefuran was reported in runoff from paddy fields Yokoyama et al. (2015). Further, these concentrations are much lower than Japanese maximum residue limits (MRLs) in representative crops (The Japan Food Chemical Research Foundation (JFCRF), 2018). MRLs are, for examples, 2 ppm of dinotefuran (DF) and 1 ppm of clothianidin (CN) in brown rice, 25 ppm of DF and 20 ppm of CN in lettuce, 15 ppm of DF and 40 ppm of CN in spinach, 15 ppm of DF and 5 ppm of CN in grapes, and 25 ppm of DF and 50 ppm of CN. Experimental concentrations seem to be realistic in relation to pesticide use in Japan.

### Field experiments

**Figure 1:**
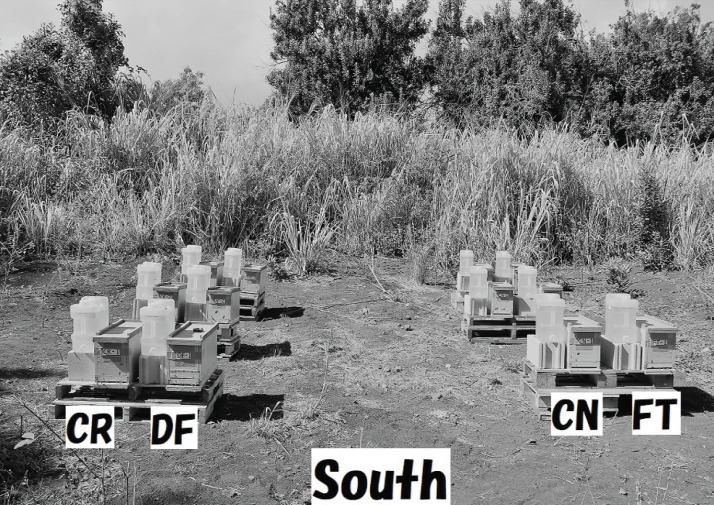
Experimental site in Maui. Twelve beehives composed of four colony groups (CR, DF, CN and FT groups), each of which have three colonies under the same concentration of the same pesticide (CR-1, CR-2, CR-3; DF-1, DF-2, DF-3; CN-1, CN-2, CN-3; FT-1, FT-2, FT-3), were sited in four columns (same pesticide) and three rows (different pesticides) facing south.

We conducted field experiments in Maui (Latitude 20°55′00.2″ N, Longitude 156°30′39.2″ W) from October 22, 2014 to July 20, 2015. The experimental site was in macadamia woods surrounded by sea and non-commercial organically-managed farmlands. Honeybees gather foods (take nectar, pollen) at any time of the year in the experimental site in Maui, Macadamia trees blooming from January to June, come into flower six times annually, and other flowers populate the woods throughout the year. Twelve beehives composed of four colony groups (CR, DF, CN and FT groups), each with three colonies administered the same concentration of the same pesticide (CR-1, CR-2, CR-3; DF-1, DF-2, DF-3; CN-1, CN-2, CN-3; FT-1, FT-2, FT-3), were sited in four columns (same pesticide) and three rows (different pesticides) facing south (Fig. 1). Each hive had three combs and a feeder for sugar syrup. An auto-feeding system from which sugar syrup was automatically and continuously fed to a small tray placed on the bottom of the inside of a hive. Ten-liter containers, containing about 14 kg of sugar syrup, were used to supply the syrup. We compared the experimental conditions of four field experiments in mid-west Japan and those of one field experiment in Maui (see Table S1). Shown in Table S1 are experimental periods, sites, objectives of each study, environmental conditions surrounding experimental sites, experimental conditions, and publications of research results.

We carefully conducted the experiment paying close attention to experimental procedures (see Table S2) for determining numbers of adult bees and capped brood. The accuracy of these measurements greatly affects the accuracy of apparent longevity calculations. Care was taken to as prevent errors caused by inappropriate experimental environment, mistakes in experimental procedures, inappropriate experimental procedures, mistakes in photography, and errors in counting.

The experiment started on October 22, 2014 with feeding of pesticide-free sugar syrup. On the next day, October 23, 2014, feeding with sugar syrup containing pesticide was provided to experimental colonies. Beekeepers advise that honeybees are apt to ingest nectar gathered from fields rather than sugar syrup fed into a hive. Syrup is likely to be stored during the blooming season. If so, any toxic effects might not be immediately observed when a pesticide is administered in sugar syrup when other food sources are available. Effects would be delayed until other food sources become scarce. We decided to terminate the experiment when all colonies failed or the end of July 2015, when few flowers are in bloom. Pesticide administration was terminated just before the spring at the end of March or the beginning of April. We assumed that pesticide exposure would be small when honeybees sought nectar instead of sugar syrup and tend to store sugar syrup without direct ingestion. Every queen used in this experiment was a sister that emerged concurrently. More details of the experiment are reported in our previous article (Yamada, Yamada & Apao, 2018).

### Counting methods of adult bees, brood, mites, and dead bees

**Figure 2:**
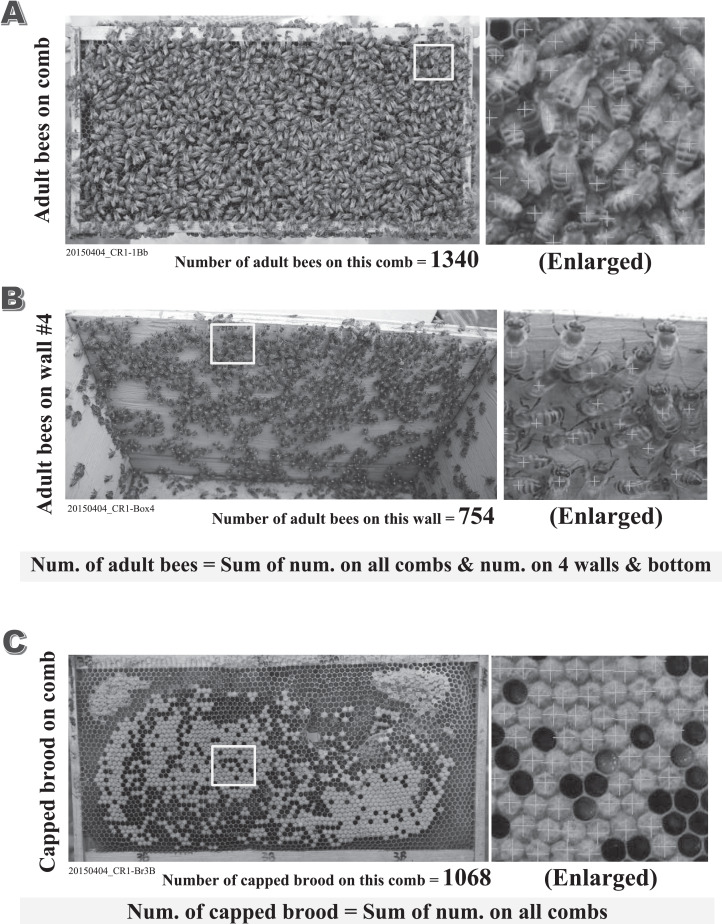
Examples of counted adult bees and capped brood. All figures (A–C) are the counted examples of the control colony “CR-1” in the experiment conducted on April 4th in 2015. (A) The counted example of adult bees on the back face comb of the first frame in CR-1. (B) The counted example of adult bees on the inside wall #4 in the hive-box of CR-1. (C) The counted example of capped brood on the back face comb of the third frame in CR-1. The total number of adult bees in each colony can be obtained by the sum of the numbers of adult bees on all combs and the numbers of adult bees on all walls and bottom in the hive-box. The total number of capped brood in each colony can be obtained by the sum of the numbers of capped brood on all combs. The numbers of adult bees and capped brood are of great importance to estimate the apparent longevity accurately. We count the numbers of adult bees and capped brood on the photograph as correctly as possible. Their numbers are roughly counted using the image processing software (the improved “Perfect Viewer 7” made by Nanosystem Corporation, Japan) after an optimal threshold is searched on each image. Their numbers are recounted accurately within an error of 3% with the photograph being enlarged to ensure overlapped bees and bees on the unsharp image. The total number of adult bees in a colony are obtained by summing up the number on every comb surface and that on the four walls and bottom in the hive. The total number of capped brood in a colony are obtained by summing up the number on every comb surface without bees.

Numbers of adult bees, capped brood and mites were accurately counted from photographs taken during the experiment (Fig. 2). Adult bees and capped brood are initially roughly counted using the image processing software “Perfect Viewer 7” (Nanosystem Corporation, Kyoto, Japan) with optimal threshold for adult bees or capped brood. Subsequently, are accurately counted by eye on enlarged images with mistakes on the roughly counted image carefully corrected (Errors may include overlaid bees, a capped honey cell, and fuzzy photographs). The adult bees were counted each side of each comb and on the inside of the hives (four walls and bottom). The sum of all the counted adult bees was used as the number of adult bees in a colony. Similarly, the sum of all the counted capped brood on all combs was used as the number of capped brood in a colony. An incompletely capped brood is regarded as a capped brood when the capped area of the cell in which a brood exists is more than 90%. The numbers obtained from photographs can be counted again at any time when in doubt. The numbers of adult bees and capped brood were recorded in a datasheet after rechecking on a counted photograph. Adult bees and capped brood were counted within a 3% error, similar to previous studies (Yamada, Yamada & Yamada, 2018a, 2018b, 2018c; Yamada, Yamada & Apao, 2018).

Mites were directly counted by summing numbers of mites on bees, inside hives (notably, on the bottom of hives) and in open cells on a comb without bees. Counting was aided by enlarging photographs using the improved Perfect Viewer 7 (image processing software) that was used to count numbers of adult bees and capped brood. Mites under wax-capped cells could not be counted. This method for counting mites does not provide an accurate count for mites, but will likely give a reasonable rough estimate under the assumption that mites can be found on bees or in open cells when they exist in capped cells. We could not find mites in every colony in this field experiment in Maui though we carefully examined every photograph enlarged on a computer display.

Dead bees were directly counted, one by one, with a pair of tweezers and then tossed out of the hive, by summing up the numbers of dead bees on a tray laid under a hive, bees in the sugar syrup feeder, and dead bees inside a hive (notably, the bottom of a hive), similar to previous studies. Counting error of dead bees is negligible.

### Mathematical model for apparent longevity

We previously proposed the following mathematical model to estimate apparent longevity using only numbers of adult bees and capped brood (Yamada, Yamada & Yamada, 2019):

The dynamic parameter, “apparent longevity”, *L*(*t*), is defined in Eq. (1), The calculation characterizes the “border” age at which an average worker bee disappears from the colony:

(1) $${a}\left( t \right) = \mathop \int \nolimits_{T_{\rm pupa}}^{T_{\rm pupa} + L\left( t \right)} u\left( {t - s} \right)p\left( {t - s} \right)ds$$

where *a*(*t*) and *u*(*t*) denote numbers of adult bees and newly capped brood, respectively, per unit time *t*, *p*(*t*) is eclosion rate of pupae capped at time *t*, and *T*_pupa_ denotes the average capped brood period, which is 12 days.

It is noteworthy that if a colony is in a steady state, where the worker bees die at the same age, *L*(*t*) represents the lifespan of an adult worker bee and may, therefore, provide an estimation of the longevity of individual bees in a normal colony. At steady state, the distribution of dying bees approximates a narrow Gaussian distribution.

To obtain apparent longevity, *L*(*t*), in Eq. (1), *a*(*t*), *u*(*t*), and *p*(*t*) are essential. *a*(*t*) and *p*(*t*) can be obtained from our measurements. Since it is difficult to obtain *u*(*t*) directly, we estimate it from Eq. (2):

(2) $$b\left( t \right) = \mathop \int \nolimits_0^{T_{\rm pupa}} u\left( {t - s} \right)\left[ {p\left( {t - s} \right)} \right]s/T_{\rm pupa}\; ds$$

where *b(t*) is the total number of capped brood at time *t*.

Note that we obtain Eq. (2) assuming that a pupa is removed immediately when it is dead in a cell before eclosion.

The combination of Eqs. (1) and (2) characterizes a colony using macroscopic quantities (total numbers of adults and capped brood), which are easily accessible. Though *a*(*t*) in Eq. (1) and *b*(*t*) in Eq. (2) are continuous, their measurements, obtained on specific measurement days are discrete in an actual experiment. Therefore, it is necessary to discretize Eqs. (1) and (2) in order to obtain apparent longevity *L*(*t*). Data time-discretization is achieved under the assumptions that the number of newly capped brood and eclosion rate is constant between two successive measurements (i.e., *u*(*t*) and *p*(*t*) have the following functional forms):

(3) $$\eqalign{& u(t) = \nu (t - T_{\rm egg}) =\cr & \left\{ {\; \matrix{ {{{\bar u}_0}\; (t < {t_0})}\; {\rm before\; starting\; the\; experiment} \cr {{{\bar u}_1}\; \left( {{t_0} \le t < {t_1}} \right)}\; {\rm from\; the\; initial\; observation\; to\; the\; first\; one} \cr {{{\bar u}_2}\; \left( {{t_1}\; \le \; t < {t_2}} \right)}\; {\rm from\; the\; first\; observation\; to\; the\; second\; one} \cr \vdots \cr {{{\bar {u} }_k}\; \left( {{t_{k - 1 }} \le t < {t_k}} \right)}\; {\rm from\; the}\; (k - 1){\rm th\; observation\; to\; the}\; k{\rm th\; one} \cr \vdots \cr {{{\bar { u} }_{N - 1}}\; \left( {{t_{N - 2}}\; \le t < {t_{N - 1}}} \right)}\; {\rm from\; the\; previous\; observation\; to\; the\; final\; one} \cr } } \right.}$$

(4) $$p\left( t \right) = \left\{ {\; \matrix{ {{{\bar p}_0}\; (t\; < \; {t_0})} \cr {{{\bar p}_1}\; \left( {{t_0}\; \le \; t\; < {\rm \; }{t_1}} \right)} \cr {{{\bar p}_2}\; \left( {{t_1}\; \le \; t\; < {\rm \; }{t_2}} \right)} \cr \vdots \cr {{{\bar p }_k}\; \left( {{t_{k\; - \; 1\; }} \le \; t\; < \; {t_k}} \right)} \cr \vdots \cr {{{\bar p}_{N\; - \; 1}}\; \left( {{t_{N\; - \; 2}}\; \le \; t\; < \; {t_{N\; - \; 1}}} \right)} \cr } } \right.$$

where *t_k_* denotes the time at the *k*th measurement. The subscript *k* takes from 0 (at the start of the experiment) to *N −* 1 (at final measurement).

Since ${\bar p_k}$ is given by the eclosion rate reported previously (Yang et al., 2012), there are *N*-variables to be determined in Eq. (2): ${\bar u_0}$, ${\bar u_2}$ …${\bar u_{N-1}}$. Using the measured number of capped brood at *t* = *t_k_*, *b*(*t_k_*), we obtain the following system of linear equations:

(5) $$b\left( {t_k} \right) = \mathop \int \nolimits_0^{T_{\rm pupa}} u\left( {t_k - s} \right)\left[ {p\left( {t_k - s} \right)} \right]s/T_{\rm pupa}\; ds\quad {\rm for}\quad k = 0, \ldots , N - 1$$

This system is easily solved using standard Gaussian elimination, which yields a set of solutions, ${\bar u_0}$, ${\bar u_2}$ …${\bar u_{N-1}}$. Then, *L*(*t*_0_),…, *L*(*t*_*N*_) are found from the discretized version of Eq. (1) by using an iterative procedure, with the values of ${\bar u_0}$, ${\bar u_2}$ …${\bar u_{N-1}}$ obtained from Eq. (5).

Symbols used in this mathematical are explained model below:

*T*_egg_ is the egg and larval period (from the time the queen lays eggs till they become a capped brood) and *T*_egg_ = 9 days for a honeybee; *T*_pupa_ is the capped brood period, mainly pupal period (from the time a brood starts to be capped till the capped brood ecloses); *T*_pupa_ = 12 days for a honeybee; *a*(*t*) is the number of adult bees; *v*(*t*) is the number of eggs laid by a queen per day in an interval between the (*k* − 1)th measurement and the *k*th; *b*(*t*) is the total number of capped brood summed from the residual and newly capped brood; *u*(*t*) is the number of newly capped brood per day in an interval between the (*k* − 1)th measurement and the *k*th at the elapsed time *t*, where *u*(*t*) = *v*(*t* − 9), because it takes 9 days for capping of honeybee eggs; *t*_*k*_ = time at the *k*th measurement, which is a discrete variable, *t* = *t*_0_, *t*_1_, …, *t*_*N* −_ 1, *t*_*N*_; *ū*_*k*_ is equal to *u*(*t*_*k*_) which is assumed to be constant between the (*k* − 1*)*th measurement and the *k*th, to obtain the value of *u*(*t*). We assume the following because capped brood hatches over 12 days for honeybees; *L*(*t*) is the apparent longevity of a colony. Few bees are lost in winter because of extremely low foraging activity, and apparent longevity is roughly equivalent to the longevity of oldest member of that group. *p*(*t*) is the probability of the brood newly capped at time *t* eclosing and developing into imagos (adult bees); ${\bar p_{k\; }}$ is *p*(*t*_*k*_) assumed to be constant between the (*k* − 1)th measurement and the *k*th, which is estimated from the eclosion rate curve of the capped brood.

We make the following assumptions to solve the above equations: An equal number of brood (larvae) are capped per day between a given observation and the next. One-twelfth of all capped brood, *b*(*k*), which are measured in the *k*th experiment will turn into new adult bees per day between the *k*th experiment and *k* + 1th experiment because new adult bees emerge from capped brood after 12 days from the day when a brood is capped. When an interval between a given observation and the next is longer than 12 days, the number of new adult bees emerging from capped brood remains the same to the next experiment. In this work, Ruby programing language was used to solve the equations in the mathematical model. Apparent longevity of a colony composed of social insects other than honeybees can be estimated using the mathematical model proposed in this paper when the life cycle of the insect is known (*T*_egg_, *T*_pupa_, and *p*(*t*) can be obtained) and the numbers of imagos and pupae can be accurately measured at given time intervals.

## Results

### Outline of long-term observations

All six neonicotinoids (dinotefuran, clothianidin)-exposed colonies failed during the experimental period. Only one of three organophosphate (fenitrothion)-exposed colonies and only one of three control (pesticide-free) colonies failed during this period. The rate of colony extinction in Maui is about the same as that in Shika. Examining the process of colony failure we found a difference between the neonicotinoid-exposed colonies and the other colonies: There were no wax-moth larvae in the six neonicotinoid-exposed colonies (DF-1, DF-2, DF-3, CN-1, CN-2, CN-3) but several wax-moth larvae were found in the control colony (CR-2) and in the fenitrothion-exposed colony (FT-1) when they failed.

### Number of adult bees

**Figure 3:**
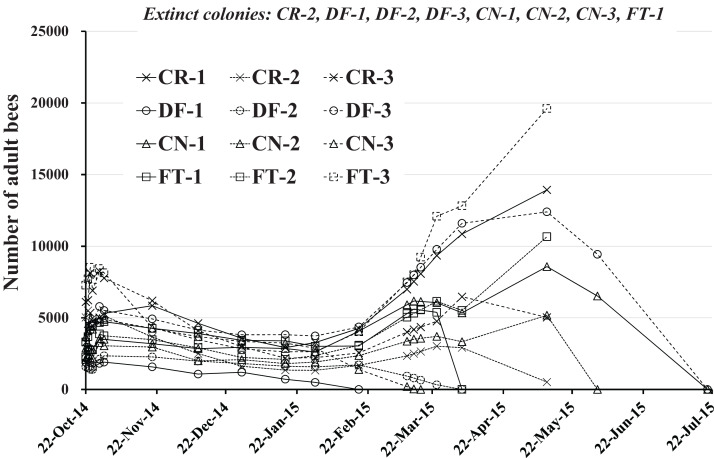
Change in the number of adult bees in Maui (Hawaii). Extinct colony and extinct date (Elapsed days from the start) are as follows. CR-2: May 11, 2015 (201 days), DF-1:February 18, 2015 (119 days), DF-2:April 4, 2015 (164 days), DF-3:July 20, 2015 (271 days), CN-1:July 20, 2015 (271 days), CN-2:June 2, 2015 (223 days), CN-3:March 17, 2015 (146 days), FT-1:April 4, 2015 (164 days).

The numbers of adult bees in colonies obtained during the study are shown in Table 1 and Fig. 3. Each colony seems to change similarly with the seasons except when a colony fails. The change in numbers of adult bees seems to correspond to the degree of blooming in Maui where few flowers bloom in January and June and many flowers are in full bloom in April and September.

**Table 1:** Number of adult bees in Maui (Hawaii).

Date	Elapse d (day)	Number of adult bees											
		CR-1	CR-2	CR-3	DF-1	DF-2	DF-3	CN-1	CN-2	CN-3	FT-1	FT-2	FT-3
22-Oct-14	0	3,287	5,031	6,109	1,903	1,580	2,199	2,890	2,094	3,658	3,317	2,261	7,277
23-Oct-14	1	4,584	5,507	6,230	2,147	1,517	2,949	2,885	2,195	4,096	3,680	2,730	7,716
24-Oct-14	2	5,341	5,327	8,131	1,889	1,405	4,130	2,809	2,212	4,385	4,407	2,737	8,517
25-Oct-14	3	4,778	4,964	6,904	1,547	1,391	4,469	2,798	2,157	4,204	4,454	2,752	7,666
28-Oct-14	6	5,017	4,665	8,244	1,814	2,116	5,788	3,553	3,272	4,949	4,669	3,870	8,431
30-Oct-14	8	5,311	5,153	7,778	1,908	2,348	5,515	3,497	3,050	4,880	4,732	3,748	8,142
20-Nov-14	29	5,845	3,663	6,200	1,576	2,272	4,923	3,189	2,960	4,259	4,259	3,476	4,319
10-Dec-14	49	4,619	2,501	4,094	1,074	1,979	4,145	2,852	1,999	3,685	3,908	2,903	3,466
29-Dec-14	68	3,569	1,619	2,978	1,202	1,810	3,818	2,935	2,080	3,349	3,427	2,233	2,837
17-Jan-15	87	2,922	1,348	2,186	711	1,620	3,823	2,855	1,806	3,243	3,389	2,098	2,628
30-Jan-15	100	3,275	1,336	2,221	495	1,580	3,735	2,614	1,917	2,871	3,009	2,369	2,641
18-Feb-15	119	4,261	1,683	2,550	0	1,714	4,361	4,074	2,361	1,388	3,032	3,087	4,024
11-Mar-15	140	6,989	2,341	4,003		933	7,454	5,911	3,367	201	5,290	5,037	7,507
14-Mar-15	143	7,534	2,498	4178		793	7,991	6,173	3,511	31	5,612	5,315	8,005
17-Mar-15	146	8,079	2,654	4352		653	8,529	6,139	3,570	0	5,543	5,566	9,231
24-Mar-15	153	9,350	3,024	4,760		326	9,783	6,060	3,709		5,381	6,150	12,091
4-Apr-15	164	10,857	2,916	6,476		0	11,600	5,350	3,334		0	5,512	12,820
11-May-15	201	13,928	516	5,033			12,398	8,573	5,179			10,660	19,609
2-June-15	223						9,433	6,523	0				
20-July-15	271						0	0					

**Note:**

Experiment started on October 22nd in 2014 and a pesticide was administered from October 23rd in 2014 to April 4th in 2015.

For extinct colonies of CR-2, DF-1, DF-2, DF-3, CN-1, CN-2, CN-3 and FT-1 extinct dates and elapsed days from the start of the experiment (October 22, 2014) until colony failure are: CR-2, May 11, 2015 and 201 days; DF-1, February 18, 2015 and 119 days; DF-2 April 4, 2015 and 164 days; DF-3, July 20, 2015 and 271 days; CN-1, July 20, 2015 and 271 days; CN-2, June 2, 2015 and 223 days; CN-3, March 17, 2015 and 271 days; FT-1, April 4, 2015 and 164 days. DF-1, DF-2, and CN-3 became extinct before April, displaying low numbers of adult bees without increased numbers of adults in the April blooming season. CN-2 became extinct at the beginning of June, showing a similar profile in adult bee numbers. DF-3 and CN-1 failed in late July after bee numbers showed a profile typical of Maui as described by P. Apao (2014) personal communication. Colony CN-1 displayed a smaller increase in bee numbers for March than typical. FT-1 failed at the beginning of April after a slight increase in the bee numbers. CR-2 became extinct in mid-May after a slight increase in bee numbers while maintaining low numbers. The surviving colonies of CR-1, CR-3, FT-2, and FT-3 showed bee number profiles typical of Maui.

### Number of capped brood

**Figure 4:**
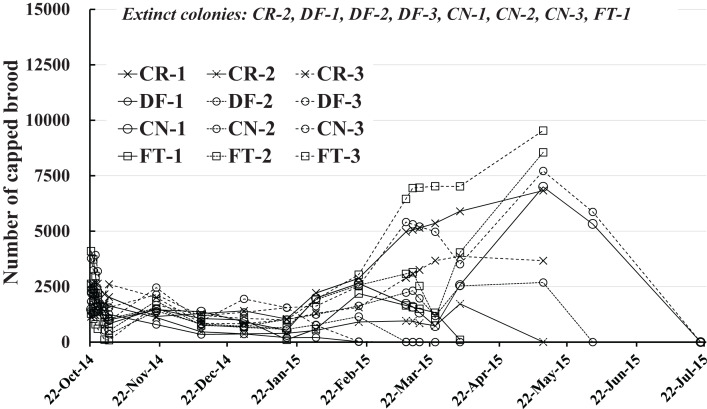
Change in the number of capped brood in Maui (Hawaii).

The number of capped brood in each colony is shown in Fig. 4 and Table 2. Capped brood showed a roughly similar change in numbers as did the number of adult bees, though numbers varied more widely: Capped brood increased rapidly from the latter half of February in 2015 after a slight decrease after the start of the experiment to the latter half of January. We speculated that flowering season and weather was intertwined in changes in numbers of adult bees and capped brood.

**Table 2:** Number of capped brood in Maui (Hawaii).

Date	Elapse d (day)	Number of capped brood											
		CR-1	CR-2	CR-3	DF-1	DF-2	DF-3	CN-1	CN-2	CN-3	FT-1	FT-2	FT-3
22-Oct-14	0	1,144	1,648	2,595	2,267	1,832	3,775	1,474	2,203	2,386	4,103	2,632	1,284
23-Oct-14	1	1,186	1,442	2,620	2,203	1,856	3,240	1,371	2,068	2,370	3,728	2,445	1,050
24-Oct-14	2	1,204	1,585	2,649	1,880	1,880	3,929	1,388	1,890	2,269	2,988	2,191	806
25-Oct-14	3	1,274	1,634	2,635	1,746	1,639	3,191	1,403	1,605	2,083	2,222	1,954	600
28-Oct-14	6	1,691	1,765	2,157	1,395	1,195	1,386	1,225	533	1,606	918	799	114
30-Oct-14	8	2,029	1,638	2,605	1,253	1,065	956	1,000	354	1,559	827	513	89
20-Nov-14	29	1,154	1,111	2,016	792	1,520	2,457	1,454	1,403	2,138	1,501	1,854	1,676
10-Dec-14	49	1,290	462	849	345	782	944	1,373	827	1,194	1,058	751	1,213
29-Dec-14	68	1,409	371	828	366	659	1,946	918	712	1,342	1,000	693	1,066
17-Jan-15	87	1,043	398	1,019	200	575	1,557	529	1,024	1,550	108	644	1,005
30-Jan-15	100	2,215	528	1,299	209	781	1,621	1,984	1,240	763	587	1,913	1,926
18-Feb-15	119	2,876	908	1,566	17	1,153	2,518	2,644	1,636	26	2,190	2,564	3,042
11-Mar-15	140	4,974	954	2,894		8	5,412	1,727	2,231	3	1,653	3,079	6,458
14-Mar-15	143	5,062	949	3,074		6	5,311	1,596	2,316	0	1,576	3,153	6,946
17-Mar-15	146	5,150	852	3,253		4	5,211	1,333	1,975	0	1,501	2,533	6,970
24-Mar-15	153	5,355	734	3,672		0	4,976	719	1,178		1,326	1,086	7,027
4-Apr-15	164	5,897	1,722	3,866		0	3,514	2,572	2,532		107	4,043	7,023
11-May-15	201	6,831	3	3,671			7,715	7,001	2,688			8,555	9,540
2-June-15	223						5,870	5,327	0				
20-July-15	271						0	0					

**Note:**

Experiment started on October 22nd in 2014 and a pesticide was administered from October 23rd in 2014 to April 4th in 2015.

### Number of dead bees

**Figure 5:**
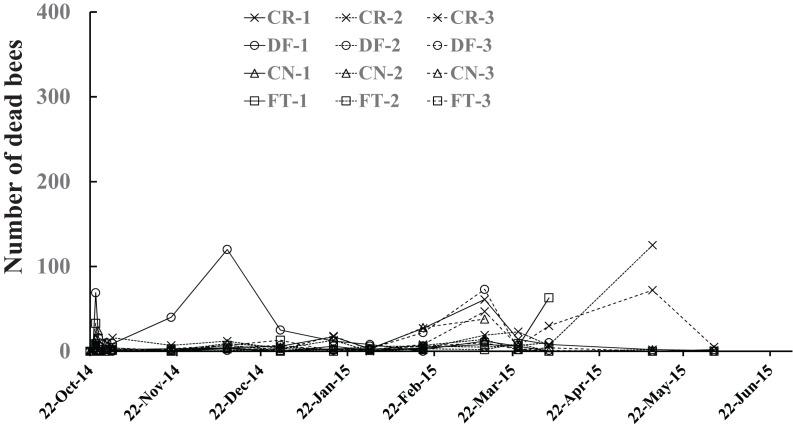
Number of dead bees in Maui (Hawaii).

The number of dead bees in a colony (Fig. 5) was obtained by summing up the numbers of dead bees on large tray laid under the hive, dead bees left in the inside of the hive, dead bees in sugar syrup, and in cells of combs. All dead bees were tossed out of the hive one by one while being counted to avoid accidentally counting dead bees as adult bees in photographs. Few dead bees were observed in colonies, at the most 125, throughout the experimental period Fig. 5.

### Intake of pesticide

In Maui, pesticide was administered in sugar syrup. Intake of the pesticide was directly obtained from the amount of sugar syrup consumed. Intake of pesticide per colony is a simple indicator of the impact of a pesticide on honeybees. However, since intake depends on the size of the colony, intake cannot always estimate the impact of a pesticide on individual honeybees. For example, pesticide impact on individual honeybees will differ between larger and smaller colonies. We proposed an estimation method for total numbers of honeybees involved in intake of pesticide throughout the experiment (Yamada, Yamada & Yamada, 2018a, 2018b). Intake per bee is calculated from the cumulative amount of pesticide consumed by a colony and the cumulative number of honeybees in a colony during the pesticide exposure period. This method seems to roughly assess the impact of a pesticide on individual honeybees. We will examine the intake of pesticide below. The details of the pesticide intake in Maui are described in Yamada, Yamada & Apao (2018).

#### Intake of pesticide consumed by a colony

**Figure 6:**
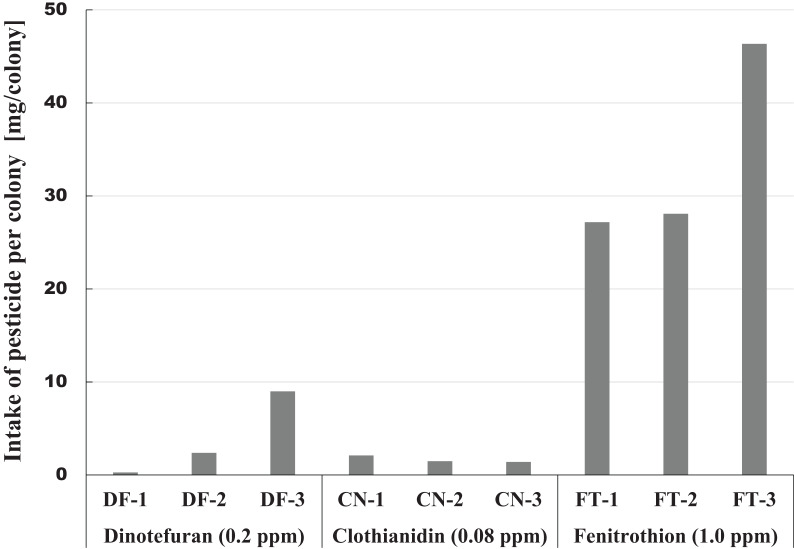
Intake of pesticide per colony during the administration period of pesticide in Maui (Hawaii).

The amount of pesticide consumed by a honeybee colony is one indicator of the magnitude of pesticide impact. This information can be easily obtained from the difference between the amount of pesticide contaminated syrup administered and the amount remaining. Figure 6 shows intake of each pesticide per colony during the pesticide administration period from the start of pesticide administration (October 23, 2014) to termination (April 4, 2015) or till the extinction of the colony whichever came first. A considerable difference was observed within the same pesticide group, and a great difference was found among different groups in pesticide intake per colony (Fig. 6). The differences within the same group of colonies may be caused by the differences in colony size or strengths of queens. Differences among groups seem to be associated with characteristics of pesticides, such as persistency and toxicity to honeybees (adult bee, brood, queen).

#### Intake of pesticides ingested by a honeybee

**Figure 7:**
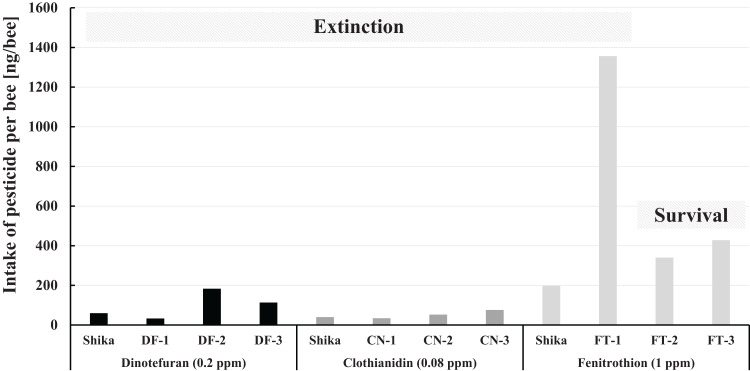
Intake of pesticide per bee during the pesticide administration period in Maui (Hawaii) and that in Shika (mid-west Japan). Shika denotes the intake of pesticide per bee during the administration period of each pesticide in Shika (mid-west Japan) and the others denote that in Maui (Hawaii).

The intake of pesticide per bee during pesticide administration in Maui in comparison with that in Shika (mid-west Japan) is illustrated in Fig. 7. The fenitrothion-exposed colony group, the dinotefuran group, and the clothianidin group are roughly ranked in descending order of the pesticide intake per bee. Examining intake of fenitrothion per bee in detail: Intake of fenitrothion per colony in FT-3 was the highest of all the fenitrothion-exposed colonies (see Fig. 6), but the intake per bee in FT-3 was much lower than in FT-1 (see Fig. 7).

One reason for the large difference in the intake between colonies and per bee seems to be that FT-3 was very active, with many more bees than the other fenitrothion-exposed colonies. Thus, a ratio of house bees that ingest sugar syrup in a hive to foraging bees becomes lower, even though the total number of adult bees is much higher. Pesticide intake per bee hence becomes less than for other FT colonies. Similar reasoning can be applied for the case of DF-3 intake of dinotefuran per bee in DF-3 whose intake of dinotefuran per colony is the most of all the dinotefuran-exposed colony become fewer than that in DF-2.

Comparing the intake of a pesticide per bee between in Maui and Shika, no differences in intake of neonicotinoid are evident. The fenitrothion-exposed colony in Shika shows lower pesticide intake per bee than the colonies in Maui, it also failed. Colony extinction in Shika is assumed not to be due to fenitrothion intake but to extraordinary circumstances such as the break of the bee-cluster in winter when the hive was opened for photography (Yamada, Yamada & Yamada, 2018c).

### Long-term change in apparent longevity

**Figure 8:**
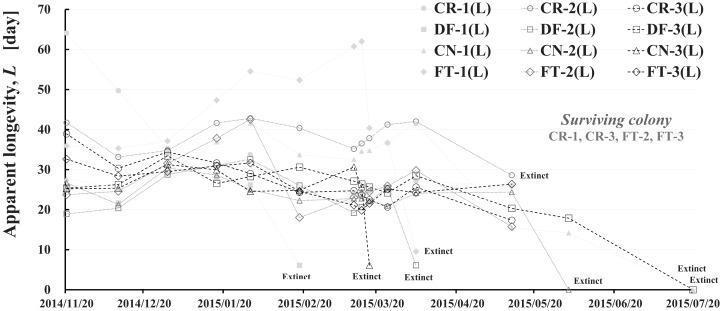
Apparent longevity of each honeybee colony in Maui (Hawaii). CR-1(L), CR-2(L) and CR-3(L) denote apparent longevity of each control (pesticide-free) colony. DF-1(L), DF-2(L) and DF-3(L) denote apparent longevity of each dinotefuran-exposed colony. CN-1(L), CN-2(L) and CN-3(L) denote apparent longevity of each clothianidin-exposed colony. FT-1(L), FT-2(L) and FT-3(L) denote apparent longevity of each fenitrothion-exposed colony.

**Figure 9:**
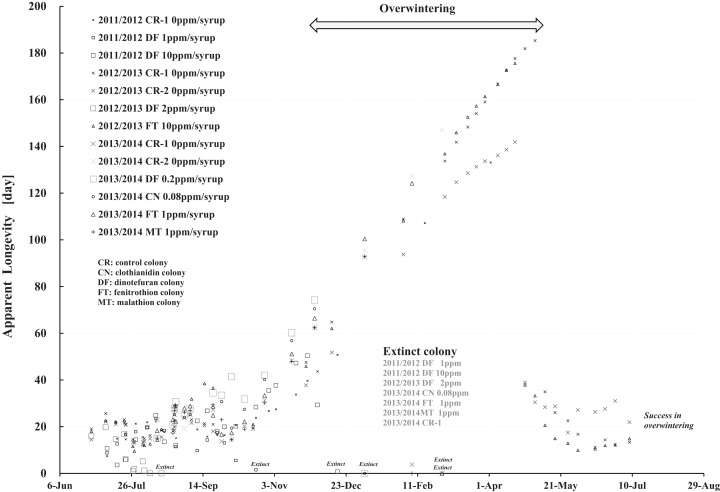
Apparent longevities of honeybee control colonies in three long-term field experiments (2011/2012, 2012/2013, 2013/2014) conducted in Shika (mid-west Japan). 2011/2012, 2012/2013 and 2013/2014 denote the long-term field experiments which were conducted from 2011 to 2012 (Yamada, Yamada & Yamada, 2018a), from 2012 to 2013 (Yamada, Yamada & Yamada, 2018b) and from 2013 to 2014 (Yamada, Yamada & Yamada, 2018c) in the mid-west of Japan (Shika), respectively. This figure is cited from Yamada, Yamada & Yamada (2019).

Apparent longevity was calculated using the numbers of adult bees (see Fig. 1) and capped brood (see Fig. 2) in each colony. Calculations from data obtained in Maui assume a constant eclosion rate (*p*) of 0.9, as judged by pesticide concentrations used and results reported by Yang et al. (2012). Figure 8 and Table 3 show long-term apparent longevity in twelve honeybee groups of three colonies each of dinotefuran-exposed, clothianidin-exposed, fenitrothion-exposed, and control. Apparent longevity varies widely over a long period while remaining roughly constant when no distinct seasonal changes occur (Fig. 8). Apparent longevities, in colonies in mid-west Japan (Shika) are substantially prolonged in winter, from six to ten fold over apparent longevity anterior to wintering (Fig. 9).

**Table 3:** Apparent longevity (L) and number of newly capped brood (u) between two successive observational dates in Maui (Hawaii).

Date	Elapsed Days	Control (pesticide-free)						Dinotefuran (0.2 ppm)						Clothianidin (0.08 ppm)						Fenitrothion (1 ppm)					
		CR-1(u) (head)	CR-1(L) (day)	CR-2(u) (head)	CR-2(L) (day)	CR-3(u) (head)	CR-3(L) (day)	DF-1(u) (head)	DF-1(L) (day)	DF-2(u) (head)	DF-2(L) (day)	DF-3(u) (head)	DF-3(L) (day)	CN-1(u) (head)	CN-1(L) (day)	CN-2(u) (head)	CN-2(L) (day)	CN-3(u) (head)	CN-3(L) (day)	FT-1(u) (head)	FT-1(L) (day)	FT-2(u) (head)	FT-2(L) (day)	FT-3(u) (head)	FT-3(L) (day)
20-Nov-14	0	101.3	64.1	97.5	41.7	177.0	38.9	69.5	25.2	133.5	18.9	215.7	25.4	127.7	27.8	123.2	26.7	187.7	25.2	131.8	35.9	162.8	23.7	147.2	32.6
10-Dec-14	20	113.3	49.7	40.6	33.2	74.5	30.3	30.3	21.7	68.7	20.4	82.9	26.3	120.6	25.3	72.6	21.3	104.8	25.3	92.9	35.3	65.9	24.6	106.5	28.4
29-Dec-14	39	123.7	35.2	32.6	34.8	72.7	34.4	32.1	34.3	57.9	28.7	170.9	33.4	80.6	29.2	62.5	30.4	117.8	31.3	87.8	37.1	60.8	31.5	93.6	29.5
17-Jan-15	58	91.6	28.3	34.9	41.6	89.5	31.7	17.6	27.9	50.5	31.1	136.7	26.5	46.4	36.9	89.9	28.6	136.1	29.9	9.5	47.3	56.5	37.8	88.2	30.9
30-Jan-15	71	194.5	33.8	46.4	42.8	114.1	29.0	18.4	26.2	68.6	32.6	142.3	28.3	174.2	41.6	108.9	25.0	67.0	24.6	51.5	54.5	168.0	42.5	169.1	31.6
18-Feb-15	90	252.5	24.8	79.7	40.4	137.5	24.3	1.5	6.1	101.2	26.0	221.1	30.6	232.1	33.7	143.6	22.3	2.3	24.8	192.3	52.4	225.1	18.0	267.1	24.6
11-Mar-15	111	436.7	24.2	83.8	35.2	254.1	24.7			0.7	19.2	475.2	27.1	151.6	32.5	195.9	22.8	0.0	30.7	145.1	60.8	270.3	23.1	567.0	21.1
14-Mar-15	114	433.3	24.4	75.7	36.5	295.6	23.6			0.0	20.6	405.0	26.4	95.9	34.6	209.7	22.8	−0.8	25.7	108.1	62.0	274.8	23.8	688.8	19.8
17-Mar-15	117	444.0	24.6	46.7	37.8	301.5	22.4			0.0	22.1	417.0	25.6	55.0	34.8	70.7	22.2	0.2	6.1	112.4	40.4	47.1	24.5	546.0	21.6
24-Mar-15	124	468.6	25.1	65.6	41.2	323.1	20.7			0.0	25.4	432.8	24.2	53.9	36.9	81.5	21.0			114.7	36.7	59.2	25.9	595.8	25.1
4-Apr-15	135	517.9	27.2	158.0	42.0	338.0	25.6			0.0	6.1	294.0	28.5	240.2	41.5	233.7	24.1			−0.7	9.5	380.0	29.8	613.2	24.3
11-May-15	172	599.8	25.9	0.3	28.6	322.3	17.4					677.4	20.3	614.7	15.5	236.0	24.4					751.1	15.8	837.6	26.4
2-June-15	194											515.4	17.9	467.7	14.2	0.0	0.0								
20-July-15	242											0.0	0.0	0.0	0.0										

**Note:**

Eclosion rate (*p*) is assumed to be 0.9. A pesticide-administration period is from the morning of October 23 in 2014 until a colony becomes extinct, but until the morning of April 4 when a colony survives until April 4.

Focusing on the seasonal changes in apparent longevity in control colonies mid-west Japan (Fig. 9), similar seasonal changes in apparent longevity are noted among the three long-term field experiments regardless of colony population: Apparent longevity begins to increase from late September, before cold weather sets in, then continues to increase through late April and suddenly drops after reaching a maximum. The seasonal changes in apparent longevity in pesticide-exposed colonies (Fig. S1) are similar to control colonies, but most of pesticide-exposed colonies become extinct during overwintering because of weakening by pesticide exposure.

Colder weather lessens oviposition of queen bees and fewer flowers in blossom reduces workload due to the decrease in labor for nursing and foraging. According to a theory that workloads on a honeybee colony affect the longevity (Bodenheimer, 1937; Fukuda & Sekiguchi, 1966; Wolf & Schmid-Hempel, 1989), it seems reasonable that increased longevity during the winter may be caused by decreased workload. In Shika, the start of increases in apparent longevity in late September when average temperature is about 20 °C is inconsistent with the workload theory because late September is a comfortable and flowery season in mid-west Japan. The workload theory also seems inconsistent with the sudden drop of apparent longevity in late April, because the change in temperature (workload) is much less sudden than the change in apparent longevity. A honeybee group with an apparent longevity of about 40 days in late April (ave. temp: ca. 13 °C) has emerged from a pupa group in mid-March (ca. 6 °C), and egg group was laid in late February (ca. 7 °C). Thus, the seasonal change in apparent longevity cannot always be explained by changes in temperature (workload). Consequently, information on longevity may be genetically programmed to vary in response to threshold environmental conditions, such as atmospheric temperature.

Apparent longevity in mid-west Japan varies over a much narrower range than the range observed in Maui. Such differences in apparent longevity between Maui and Shika seem to reflect differences in the weather. In Shika, honeybees gather no food in winter and food is scarce in summer. However, in Maui, an adequate food supply makes it possible for queen bees to oviposit at any time as needed, and prolonging apparent longevity to maintain the colony is unnecessary. Bees gather food all year.

## Discussion

### Impact of temperature and precipitation on a honeybee

The impact of difference in climate on a honeybee colonies was examined for colonies in Maui and Shika, mid-west Japan based on monthly changes in temperature and precipitation (see Fig. S2). Temperature changes are less extreme throughout the year in Maui (nearly constant) than in Shika and the precipitation is much lower in Maui (Fig. S2). Maximum temperature in Maui ranges from about 27 to 32 °C. Honeybee foraging activity remains low at below 25 °C, and increases sharply from about 25 to about 30 °C, a maximum at about 30 °C is maintained until about 32 °C. Above 32 °C, foraging activity begins to decrease (Tesfay, 2009). Honeybee colonies in Maui seem to maintain high levels of foraging activity throughout the year. Foraging activity shows a tendency to become low when relative humidity is higher than 50% (Tesfay, 2009).

Maximum daily temperature in Shika ranges from about 7 °C to 31 °C and precipitation in Shika is much higher. Total foraging activity for the year seems to be much higher in Maui than in Shika. This means that honeybees in Maui ingest lower concentrations of pesticide in dilute sugar syrup in favor of pesticide-free nectar collected from organically-grown macadamia woods. The concentration of pesticide administered into honeybee colonies in Maui experiment was the same as for colonies at Shika, yet most of the colonies in Maui experiment lived longer. The delay of extinction in honeybee colonies exposed to a pesticide in Maui can be explained as: A honeybee colony stores pesticide, administered through sugar syrup, in comb-cells as honey mixed with pesticide-free nectar collected from organically-grown macadamia woods. The colony continues to take in honey with lower concentrations of pesticide over a long period. Lower pesticide concentrations in honey in Maui delays pesticide effect and queen bees lay more eggs, and worker bees are more active. As a result, the colony in Maui can outlive that in Shika.

### Effect of number of newly capped brood on apparent longevity

**Figure 10:**
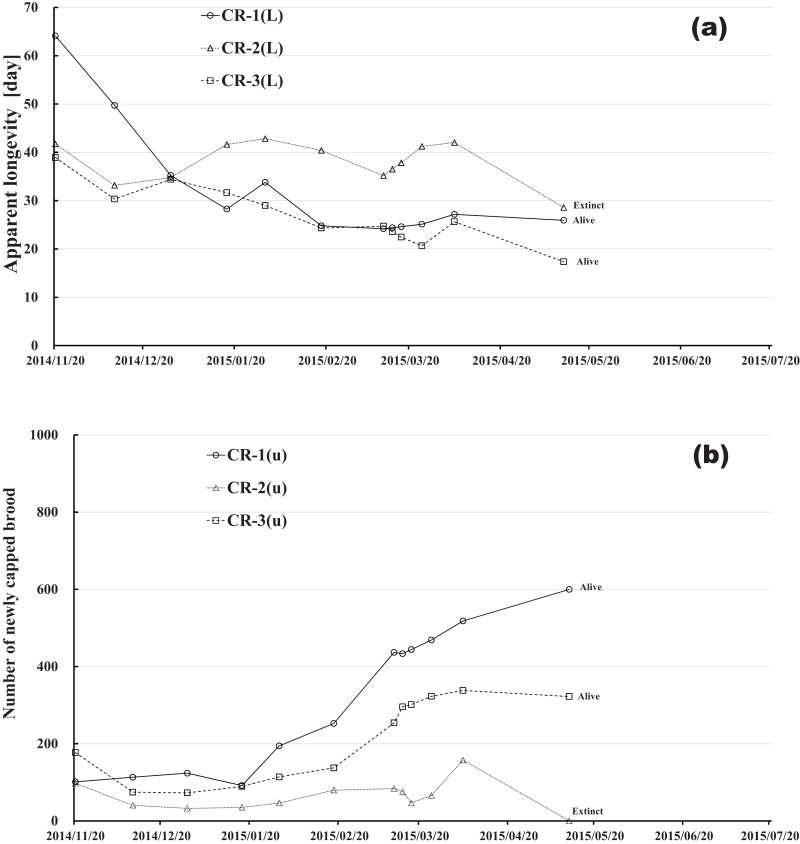
Control colony in Maui (Hawaii) where no pesticide was administered. (A) Apparent longevity. (B) Number of newly capped brood between two successive observational dates.

**Figure 11:**
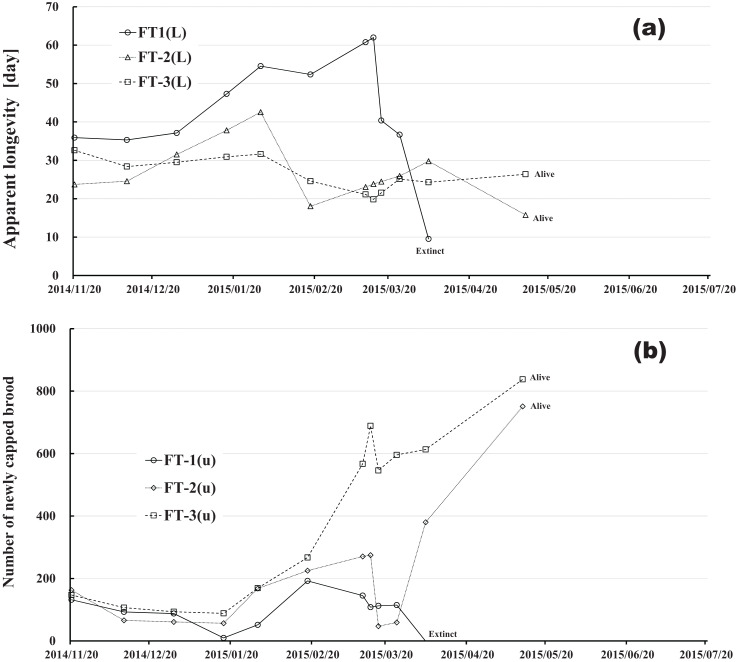
Fenitrothion-exposed colony in Maui (Hawaii). (A) Apparent longevity. (B) Number of newly capped brood between two successive observational dates.

**Figure 12:**
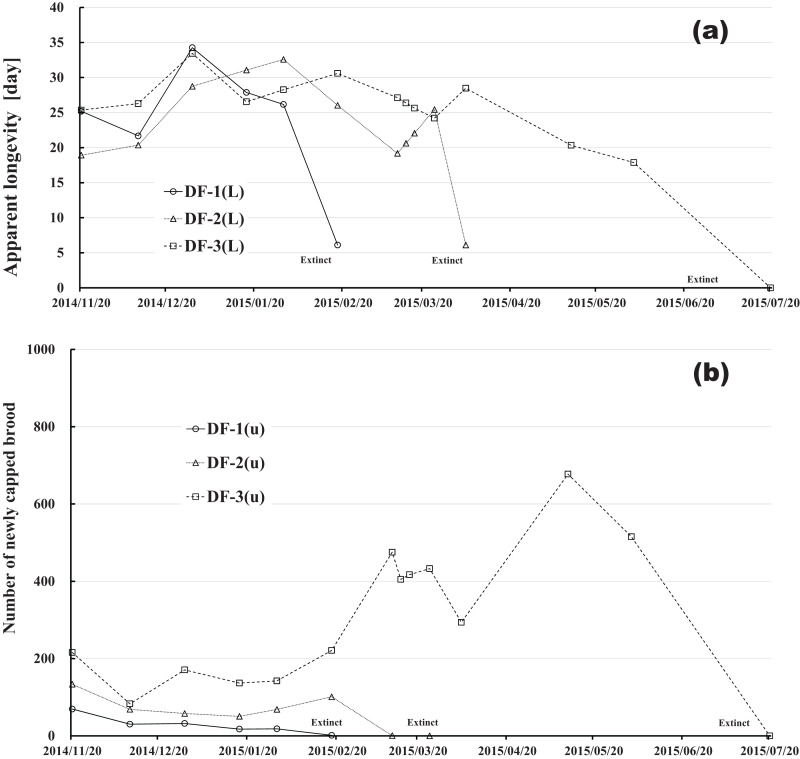
Dinotefuran-exposed colony in Maui (Hawaii). (A) Apparent longevity. (B) Number of newly capped brood between two successive observational dates.

**Figure 13:**
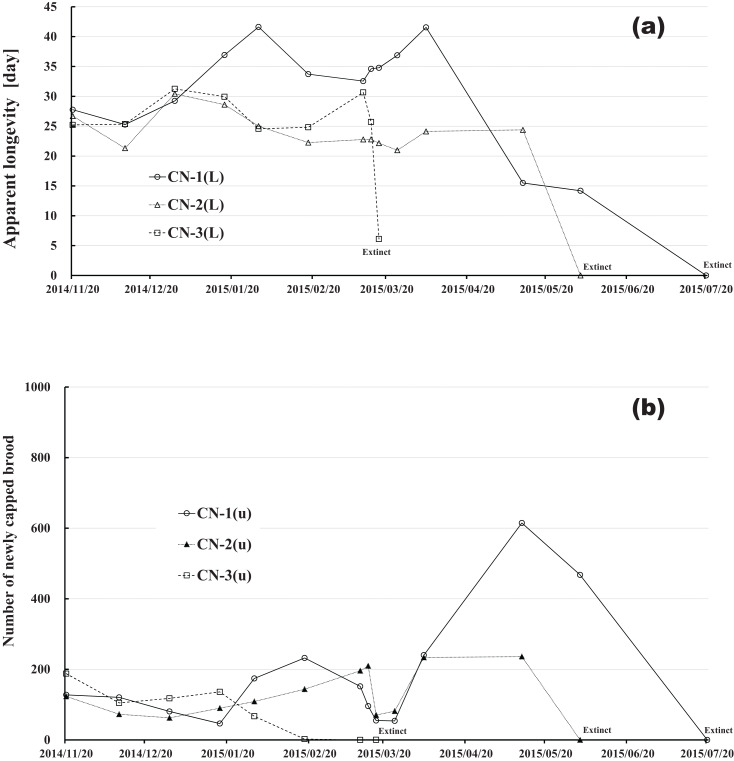
Clothianidin-exposed colony in Maui (Hawaii). (A) Apparent longevity. (B) Number of newly capped brood between two successive observational dates.

Changes in apparent longevity and numbers of newly capped brood have interesting common features between control colonies and fenitrothion-exposed colonies. Apparent longevity in the colonies (CR-2, FT-1), which has become extinct, is highest among all colonies in the same group, and the number of newly capped brood in the extinct colonies is lowest among all colonies in the same group (see Figs. 10 and 11). Such features in the neonicotinoid-exposed colony groups (dinotefuran-exposed colony group and clothianidin-exposed colony group) are not evident (Figs. 12 and 13). These features suggest that causes leading to extinction in CR-2 (Fig. 10) and FT-1 (Fig. 11) may be similar. Still, they may be different from those in DF-1, DF-2 and DF-3 of the dinotefuran (neonicotinoid)-exposed (Fig. 12) and CN-1, CN-2 and CN-3 of the clothianidin (neonicotinoid)-exposed groups (Fig. 13).

The apparent longevities in CR-2 and FT-1 seem to be prolonged by general physiological functions to maintain smaller colonies. However, neonicotinoid (dinotefuran, clothianidin)-exposure do not exhibit such general physiological phenomena. Further, they show abnormal changes in apparent longevity judging from the relationship between apparent longevity and the number of newly capped brood. For example, apparent longevity in DF-1 is nearly equal to the others in the dinotefuran-exposed colony group, though the number of newly capped brood in DF-1 is the smallest (see Fig. 12), and the apparent longevity in CN-1 is the longest in the clothianidin-exposed colony group, though the number of newly capped brood in CN-1 is the largest (Fig. 13). Such effects initially seem incoherent, but can be explained by the relationship between apparent longevity and the number of newly capped brood. These results indicate that neonicotinoid pesticides, such as dinotefuran and clothianidin, may impede normal physiological functions.

### Comparison of Maui (Hawaii) and Shika (mid-west Japan)

Comparing Fig. 8 with Fig. 9, a few striking differences in the long-term changes in apparent longevity are evident between Maui, USA and Shika, Japan. One noticeable difference is the nearly constant average apparent longevity throughout all seasons in Maui versus extremely large changes in apparent longevity between, before, and after overwintering in Shika. Another is much wider variations and more dramatic fluctuations in apparent longevity in Maui than those in Shika. A third difference is much wider variations in the period from experiment commencement to colony extinction in Maui compared to Shika.

One reason that average apparent longevity remains approximately constant over all seasons in Maui, as distinct from Shika where it increases rapidly in winter and drops off drastically at the beginning of spring, may weather differences between the two regions. The year-round genial climate of Maui does not require lengthening apparent longevity because queens can oviposit at any time and the honeybees can obtain food throughout the year. In contrast, four distinct seasons in Shika make it necessary for apparent longevity to lengthen to allow colonies to survive cold winters when queen bees cannot oviposit.

The larger variations and fluctuations of the apparent longevity in Maui are assumed to reflect a hospitable environment where food is abundant and the climate is comfortable all through the year. Since queen bees can oviposit whenever apparent longevity can hardly be influenced by seasons. Judging from the number of newly capped brood, a detailed examination shows that queen bees seem to oviposit abundantly in spring even in seasonless Maui. In cases where little need exists to control longevity, apparent longevity seems to vary and fluctuate widely, though the annual rate of change is much less than that in temperate regions. Adjustment of apparent longevity is necessary in cases of certain particular conditions, such as swarming and the death of a queen. Such adjustment is evident even in Maui. Compared to Shika, a year-round, winterless, and comfortable climate leads to greater effects on apparent longevity for individual colonies brought on by swarming and queen bee death.

Similarly, wider variations in the period from the start of experiment to colony extinction may also be caused by the genial climate of Maui. Abundant nectar taken from the surrounding natural environment by honeybees is stored as honey in comb-cells after being mixed with sugar syrup containing a pesticide. Pesticide concentrations and toxicity are thus reduced in stored honey. As a larger colony generally has a larger number of foragers, the colony can get more food, resulting in lower pesticide levels in the stored honey. Differences in colony size thus make a difference in the toxicity of stored honey, resulting in differences in the period from the start of the experiment to colony extinction. Further, as honeybees prefer naturally obtained nectar to sugar syrup, the differences activity of foraging bees between colonies will widen the gap between the start of the experiment to colony extinction and compared with Shika colony failure. The above narrative can be applied to honey stored in comb-cells containing persistent pesticides such as neonicotinoids, but not to honey containing more labile pesticides such as organophosphates.

### Effect of pesticide concentration on bee colonies in the field

Supposing that the intake of the active ingredient (AI) per bee in a high-concentration pesticide for a short period is equal to that in a low concentration pesticide for a long period of time in a field experiment, which will be more likely to cause hive collapse? The effect will depend on the characteristics of the pesticide. If a pesticide is effective for a shorter duration like organophosphates, intake of AI per bee for high-concentration pesticide will cause colony failure more quickly than the same pesticide at a low concentration. If a pesticide is effective for a longer duration, like neonicotinoids, long-term intake of the AI per bee in at low concentration will produce almost the same adverse effect as the short term intake at high-concentration when cumulative total intakes are equal. The impact of a low concentration pesticide for a persistent neonicotinoid that requires a long period to show toxicity in a colony may show the same impact in a shorter time when present at higher concentrations. Long-term pesticide intake tests for low concentrations of pesticide may be replaced by equivalent short term intake tests at higher concentration.

Examining intakes of a pesticide per colony and those per bee in five field experiments (2010, 2011/2012, 2012/2013, 2013/2014, 2014/2015), showed considerable variability (Table 4). This variability for neonicotinoid intake seems to be independent of pesticide concentration. Considering metabolism and period of intake, the lower the pesticide concentration, the more intake of a pesticide per bee until colony extinction occurs. The intake per bee, however, does not always decrease with pesticide concentration. Comparing intake of a neonicotinoid per bee in the same experimental period, intakes per bee are about the same for the same vehicle (sugar syrup or pollen paste) through which a neonicotinoid is administered.

**Table 4:** The intakes of a pesticide per colony or those per bee in five long-term field experiments (2010, 2011/2012, 2012/2013, 2013/2014, 2014/2015).

Description	Conditions of pesticide administration			Intake of pesticide per colony in the pesticide-administration period (mg/colony)								Intake of pesticide per bee in the pesticide-administration period (ng/bee)						
	Kind of pesticide	Conc. (ppm)	Kind of vehicle	Experimental site in Shika, Japan					in Maui, Hawaii			Experiment site in Shika, Japan				in Maui, Hawaii		
				2010^(1)^		2011/2012^(2)^	2012/2013^(3)^	2013/2014^(4)^	2014/2015^(5)^			2010^(1)^	2011/2012^(2)^	2012/2013^(3)^	2013/2014^(4)^	2014/2015^(5)^		
DF-0.2 ppm-SS	Dinotefuran	0.2	Sugar syrup (SS)					0.514	0.2764	2.381	8.9848				59.69	32.95	183.14	113.2
DF-0.565 ppm-PP	Dinotefuran	0.565	Pollen paste (PP)			1.8692							60.73					
DF-1 ppm-SS	Dinotefuran	1	SS			4.208							310.7					
DF-1 ppm-SS&PP	Dinotefuran	1 & 0.5	SS & PP	9.500(SS)+0.616(PP)	10.116							349.8						
DF-2 ppm-SS	Dinotefuran	2	SS				1.55							94.06				
DF-2 ppm-SS&PP	Dinotefuran	2 & 1	SS & PP	9.691(SS)+0.540(PP)	10.231							310						
DF-5.65 ppm-PP	Dinotefuran	5.65	PP			0.5257							65.08					
DF-10 ppm-SS	Dinotefuran	10	SS			1.9							290.3					
DF-10 ppm-SS&PP	Dinotefuran	10 & 5	SS & PP	6.333(SS)+0.500(PP)	6.833							212						
CN-0.08 ppm-SS	Clothianidin	0.08	SS					0.4016	2.097	1.4825	1.4058				39.64	34.23	52.47	76.03
CN-0.4 ppm-SS	Clothianidin	0.4	SS															
CN-0.4 ppm-SS&PP	Clothianidin	0.4 & 0.2	SS & PP	3.724(SS)+0.207(PP)	3.931													
CN-0.8 ppm-SS&PP	Clothianidin	0.8 & 0.4	SS & PP	3.954(SS)+0.203(PP)	4.157													
CN-4 ppm-SS	Clothianidin	4	SS															
CN-4 ppm-SS&PP	Clothianidin	4 & 2	SS & PP	2.533(SS)+0.200(PP)	2.733													
																		
FT-1 ppm-SS	Fenitrothion	1	SS					3.86	27.18	*28.08*	*46.338*				197.09	1356.3	*339.98*	*427.96*
FT-10 ppm-SS	Fenitrothion	10	SS				*17.07*							*862.5*				
																		
MT-1 ppm-SS	Malathion	1	SS					6.75							429.29			

**Notes:**

(1) Yamada, Yamada & Wada (2012).

(2) Yamada, Yamada & Yamada (2018a).

(3) Yamada, Yamada & Yamada (2018b).

(4) Yamada, Yamada & Yamada (2018c).

(5) Yamada, Yamada & Apao (2018).

All numbers except those in italic style denote the pesticide intake in a honeybee colony which became extinct till the finish of experiment.

Numbers in italic style denote the pesticide intake in a honeybee colony which survived at the finish of experiment.

The scattering in intake among experiments seems to result from a difference in intake route (the difference of the pesticide impact between toxic sugar syrup and toxic pollen paste on a bee colony), differences between the amount of pesticide administered and amount of pesticide ingested by honeybees, measurement errors in numbers of individuals ingesting the pesticide, differences in environmental conditions among five field experiments—weather, flowering period, degree of environmental pollution by pesticides, and attacks by predators such as Japanese giant hornets, etc. Given the variability of neonicotinoid intake in field experiments, a higher concentration of neonicotinoid will likely allow data to be obtained in a shorter time than required for a low concentration study.

## Conclusions

To examine the long-term impact of neonicotinoids, dinotefuran and clothianidin, and the organophosphate, fenitrothion, on honeybee colonies, an experiment was conducted from late October in 2014 to late July in 2015. Pesticides in sugar syrup were administered to three colonies at one-five hundredths of recommended concentrations for stink bugs extermination on farmlands. Concentrations of dinotefuran, clothianidin, and fenitrothion were 0.2 ppm, 0.08 ppm and 1 ppm in sugar syrup, respectively. All six neonicotinoid (dinotefuran, clothianidin)-exposed colonies became extinct during the experiment. One of the three organophosphate (fenitrothion)-exposed colonies and one of the three control colonies failed. The findings obtained from the experiment in Maui with no mites provides evidence to support the finding obtained in Shika (mid-west Japan) that neonicotinoids, rather than mites and organophosphates, are the main cause of colony failure (Yamada, Yamada & Yamada, 2018b, 2018c).

Apparent longevity of colonies was estimated using numbers of adult bees and capped brood using a mathematical model proposed previously (Yamada, Yamada & Yamada, 2019). Comparing the seasonal change in apparent longevity in Maui, without cold winter and mites, with that in Shika (mid-west Japan) where both distinct seasons and mites occur, clear differences were discovered: Apparent longevity in a honeybee colonies remains approximately constant during Maui winters and throughout the year. In Shika, it dramatically increases more than six-fold during winter with a comparatively small variation. Differences in apparent longevity between Maui and Shika can possibly be attributed to differences in environmental factors such as climate and food. Year-round blossoms in Maui provide food (nectar, pollen) for honeybees whenever necessary. For example, apparent longevity may increase in individual colonies in response to less ovipositing by a senescent queen or decrease by greater ovipositing by a fecund queen. The irrelevance of the seasonal control results in wide variations in longevity due to particular hive conditions. In a perpetually hospitable colony environment, apparent longevity is sensitive to small changes in conditions.

Examining the long-term changes in apparent longevity that seem at first incoherent, we found differences in apparent longevity between the neonicotinoid (dinotefuran, clothianidin)-exposed colonies and organophosphate (fenitrothion)-exposed colonies. The organophosphate-exposed colony that became extinct exhibits the longest apparent longevity and the fewest number of newly capped brood just as of the control colony that also failed. Lengthening longevity when few brood are produced is reasonable to maintain the colony from a physiological point of view. This increase in apparent longevity in neonicotinoid-exposed colonies cannot be found when numbers of newly capped brood are less. Consequently, a neonicotinoid pesticide may inhibit normal physiological function.

## Supplemental Information

10.7717/peerj.9505/supp-1 Supplemental Information 1 Supplementary file 1 for Figure 5.

10.7717/peerj.9505/supp-2 Supplemental Information 2 Supplementary file 2 for figure-6.

10.7717/peerj.9505/supp-3 Supplemental Information 3 Supplementary file 3 for figure-7.

10.7717/peerj.9505/supp-4 Supplemental Information 4 Supplementary file 4 for figure-8, 10, 11, 12 and 13.

10.7717/peerj.9505/supp-5 Supplemental Information 5 Supplementary file 5 for figure-9.

10.7717/peerj.9505/supp-6 Supplemental Information 6 Data source of Figure S1.

10.7717/peerj.9505/supp-7 Supplemental Information 7 Data source of Figure S2.

10.7717/peerj.9505/supp-8 Supplemental Information 8 Supplementary figures and tables (Tables S1 & S2 and Figures S1, S2 & S3).

## Additional Information and Declarations

### Data Availability

The following information was supplied regarding data availability: The raw measurements are available as Supplemental Files.
