# Supplementary material for: Comparison of long-term changes in size and longevity of bee colonies in mid-west Japan and Maui with and without exposure to pesticide, cold winters, and mites
Source: PeerJ. 2020 Jul 28;8:e9505. doi: 10.7717/peerj.9505 (PMC7394064; doi:10.7717/peerj.9505)
Supplement: Supplemental Information 1 [file peerj-08-9505-s001.docx]

Data file for Figure 5

| **Date** | **CR-1** | **CR-2** | **CR-3** | **DF-1** | **DF-2** | **DF-3** | **CN-1** | **CN-2** | **CN-3** | **FT-1** | **FT-2** | **FT-3** |
| --- | --- | --- | --- | --- | --- | --- | --- | --- | --- | --- | --- | --- |
| **22-Oct-14** | **0** | **0** | **0** | **0** | **0** | **0** | **0** | **0** | **0** | **0** | **0** | **0** |
| **23-Oct-14** | **0** | **0** | **0** | **0** | **0** | **0** | **0** | **0** | **0** | **0** | **0** | **0** |
| **24-Oct-14** | **4** | **3** | **5** | **69** | **4** | **7** | **15** | **14** | **5** | **9** | **4** | **33** |
| **25-Oct-14** | **2** | **0** | **2** | **16** | **6** | **7** | **0** | **1** | **24** | **2** | **2** | **5** |
| **28-Oct-14** | **0** | **10** | **0** | **10** | **0** | **0** | **3** | **0** | **11** | **2** | **0** | **0** |
| **30-Oct-14** | **1** | **16** | **2** | **9** | **0** | **2** | **1** | **1** | **4** | **2** | **1** | **4** |
| **20-Nov-14** | **1** | **7** | **3** | **40** | **0** | **2** | **0** | **1** | **0** | **3** | **0** | **1** |
| **10-Dec-14** | **4** | **12** | **7** | **120** | **1** | **4** | **3** | **9** | **4** | **4** | **7** | **7** |
| **29-Dec-14** | **6** | **3** | **2** | **25** | **3** | **7** | **0** | **5** | **1** | **0** | **1** | **13** |
| **17-Jan-15** | **17** | **13** | **18** | **12** | **2** | **1** | **3** | **2** | **3** | **6** | **2** | **0** |
| **30-Jan-15** | **3** | **0** | **2** | **8** | **6** | **3** | **3** | **2** | **2** | **1** | **1** | **2** |
| **18-Feb-15** | **27** | **3** | **8** | **0** | **6** | **22** | **2** | **7** | **28** | **4** | **3** | **6** |
| **12-Mar-15** | **61** | **19** | **47** |  | **5** | **73** | **12** | **14** | **38** | **8** | **2** | **10** |
| **24-Mar-15** | **14** | **23** | **6** |  | **7** | **2** | **6** | **2** |  | **6** | **9** | **2** |
| **4-Apr-15** | **8** | **7** | **30** |  | **10** | **1** | **0** | **0** |  | **63** | **0** | **4** |
| **11-May-15** | **2** | **125** | **72** |  |  | **0** | **0** | **2** |  |  | **0** | **0** |
| **2-Jun-15** | **1** |  | **5** |  |  | **0** | **2** |  |  |  | **0** | **0** |
