# Supplementary material for: Comparison of long-term changes in size and longevity of bee colonies in mid-west Japan and Maui with and without exposure to pesticide, cold winters, and mites
Source: PeerJ. 2020 Jul 28;8:e9505. doi: 10.7717/peerj.9505 (PMC7394064; doi:10.7717/peerj.9505)
Supplement: Supplemental Information 2 [file peerj-08-9505-s002.docx]

Data file for Figure 6

|  | **Dinotefuran (0.2 ppm)** | | | **Clothianidin (0.08 ppm)** | | | **Fenitrothion (1.0 ppm)** | | |
| --- | --- | --- | --- | --- | --- | --- | --- | --- | --- |
|  | **DF-1** | **DF-2** | **DF-3** | **CN-1** | **CN-2** | **CN-3** | **FT-1** | **FT-2** | **FT-3** |
| **Total intake [mg]** | **0.2754** | **2.381** | **8.9898** | **2.097** | **1.4825** | **1.4058** | **27.18** | **28.08** | **46.338** |
