# Supplementary material for: Comparison of long-term changes in size and longevity of bee colonies in mid-west Japan and Maui with and without exposure to pesticide, cold winters, and mites
Source: PeerJ. 2020 Jul 28;8:e9505. doi: 10.7717/peerj.9505 (PMC7394064; doi:10.7717/peerj.9505)
Supplement: Supplemental Information 3 [file peerj-08-9505-s003.docx]

Data file for Figure 7

|  | **Dinotefuran (0.2 ppm)** | | | | **Clothianidin (0.08 ppm)** | | | | **Fenitrothion (1 ppm)** | | | | **MT-1ppm** |
| --- | --- | --- | --- | --- | --- | --- | --- | --- | --- | --- | --- | --- | --- |
|  | **Shika** | **DF-1** | **DF-2** | **DF-3** | **Shika** | **CN-1** | **CN-2** | **CN-3** | **Shika** | **FT-1** | **FT-2** | **FT-3** | **Shika** |
| **Intake of pesticide [ng/bee]** | **59.69** | **32.95** | **183.14** | **113.2** | **39.64** | **34.23** | **52.47** | **76.03** | **197.09** | **1356.3** | **339.98** | **427.96** | **429.29** |
