# Supplementary material for: Comparison of long-term changes in size and longevity of bee colonies in mid-west Japan and Maui with and without exposure to pesticide, cold winters, and mites
Source: PeerJ. 2020 Jul 28;8:e9505. doi: 10.7717/peerj.9505 (PMC7394064; doi:10.7717/peerj.9505)
Supplement: Supplemental Information 4 [file peerj-08-9505-s004.docx]

Data file for Figures 8, 10, 11, 12 and 13

| **Date** | **Elapsed Days** | **CR-1(u)** | **CR-1(L)** | **CR-2(u)** | **CR-2(L)** | **CR-3(u)** | **CR-3(L)** | **DF-1(u)** | **DF-1(L)** | **DF-2(u)** | **DF-2(L)** | **DF-3(u)** | **DF-3(L)** | **CN-1(u)** | **CN-1(L)** | **CN-2(u)** | **CN-2(L)** | **CN-3(u)** | **CN-3(L)** | **FT-1(u)** | **FT-1(L)** | **FT-2(u)** | **FT-2(L)** | **FT-3(u)** | **FT-3(L)** |
| --- | --- | --- | --- | --- | --- | --- | --- | --- | --- | --- | --- | --- | --- | --- | --- | --- | --- | --- | --- | --- | --- | --- | --- | --- | --- |
| **20-11-14** | **0** | **101.3** | **64.1** | **97.5** | **41.7** | **177.0** | **38.9** | **69.5** | **25.2** | **133.5** | **18.9** | **215.7** | **25.4** | **127.7** | **27.8** | **123.2** | **26.7** | **187.7** | **25.2** | **131.8** | **35.9** | **162.8** | **23.7** | **147.2** | **32.6** |
| **10-12-14** | **20** | **113.3** | **49.7** | **40.6** | **33.2** | **74.5** | **30.3** | **30.3** | **21.7** | **68.7** | **20.4** | **82.9** | **26.3** | **120.6** | **25.3** | **72.6** | **21.3** | **104.8** | **25.3** | **92.9** | **35.3** | **65.9** | **24.6** | **106.5** | **28.4** |
| **29-12-14** | **39** | **123.7** | **35.2** | **32.6** | **34.8** | **72.7** | **34.4** | **32.1** | **34.3** | **57.9** | **28.7** | **170.9** | **33.4** | **80.6** | **29.2** | **62.5** | **30.4** | **117.8** | **31.3** | **87.8** | **37.1** | **60.8** | **31.5** | **93.6** | **29.5** |
| **17-1-15** | **58** | **91.6** | **28.3** | **34.9** | **41.6** | **89.5** | **31.7** | **17.6** | **27.9** | **50.5** | **31.1** | **136.7** | **26.5** | **46.4** | **36.9** | **89.9** | **28.6** | **136.1** | **29.9** | **9.5** | **47.3** | **56.5** | **37.8** | **88.2** | **30.9** |
| **30-1-15** | **71** | **194.5** | **33.8** | **46.4** | **42.8** | **114.1** | **29.0** | **18.4** | **26.2** | **68.6** | **32.6** | **142.3** | **28.3** | **174.2** | **41.6** | **108.9** | **25.0** | **67.0** | **24.6** | **51.5** | **54.5** | **168.0** | **42.5** | **169.1** | **31.6** |
| **18-2-15** | **90** | **252.5** | **24.8** | **79.7** | **40.4** | **137.5** | **24.3** | **1.5** | **6.1** | **101.2** | **26.0** | **221.1** | **30.6** | **232.1** | **33.7** | **143.6** | **22.3** | **2.3** | **24.8** | **192.3** | **52.4** | **225.1** | **18.0** | **267.1** | **24.6** |
| **11-3-15** | **111** | **436.7** | **24.2** | **83.8** | **35.2** | **254.1** | **24.7** |  |  | **0.7** | **19.2** | **475.2** | **27.1** | **151.6** | **32.5** | **195.9** | **22.8** | **0.0** | **30.7** | **145.1** | **60.8** | **270.3** | **23.1** | **567.0** | **21.1** |
| **14-3-15** | **114** | **433.3** | **24.4** | **75.7** | **36.5** | **295.6** | **23.6** |  |  | **0.0** | **20.6** | **405.0** | **26.4** | **95.9** | **34.6** | **209.7** | **22.8** | **-0.8** | **25.7** | **108.1** | **62.0** | **274.8** | **23.8** | **688.8** | **19.8** |
| **17-3-15** | **117** | **444.0** | **24.6** | **46.7** | **37.8** | **301.5** | **22.4** |  |  | **0.0** | **22.1** | **417.0** | **25.6** | **55.0** | **34.8** | **70.7** | **22.2** | **0.2** | **6.1** | **112.4** | **40.4** | **47.1** | **24.5** | **546.0** | **21.6** |
| **24-3-15** | **124** | **468.6** | **25.1** | **65.6** | **41.2** | **323.1** | **20.7** |  |  | **0.0** | **25.4** | **432.8** | **24.2** | **53.9** | **36.9** | **81.5** | **21.0** |  |  | **114.7** | **36.7** | **59.2** | **25.9** | **595.8** | **25.1** |
| **04-4-15** | **135** | **517.9** | **27.2** | **158.0** | **42.0** | **338.0** | **25.6** |  |  | **0.0** | **6.1** | **294.0** | **28.5** | **240.2** | **41.5** | **233.7** | **24.1** |  |  | **-0.7** | **9.5** | **380.0** | **29.8** | **613.2** | **24.3** |
| **11-5-15** | **172** | **599.8** | **25.9** | **0.3** | **28.6** | **322.3** | **17.4** |  |  |  |  | **677.4** | **20.3** | **614.7** | **15.5** | **236.0** | **24.4** |  |  |  |  | **751.1** | **15.8** | **837.6** | **26.4** |
| **02-6-15** | **194** |  |  |  |  |  |  |  |  |  |  | **515.4** | **17.9** | **467.7** | **14.2** | **0.0** | **0.0** |  |  |  |  |  |  |  |  |
| **20-7-15** | **242** |  |  |  |  |  |  |  |  |  |  | **0.0** | **0.0** | **0.0** | **0.0** |  |  |  |  |  |  |  |  |  |  |

Note: Ecolosion rate (*p* ) is assumed to be 0.9.
