# Supplementary material for: Comparison of long-term changes in size and longevity of bee colonies in mid-west Japan and Maui with and without exposure to pesticide, cold winters, and mites
Source: PeerJ. 2020 Jul 28;8:e9505. doi: 10.7717/peerj.9505 (PMC7394064; doi:10.7717/peerj.9505)
Supplement: Supplemental Information 5 [file peerj-08-9505-s005.docx]

Data file for Figure 9

| **Date** | **2011/2012** | | | | | **2012/2013** | | | | **2013/2014** | | | | | |
| --- | --- | --- | --- | --- | --- | --- | --- | --- | --- | --- | --- | --- | --- | --- | --- |
|  | **CR-1 0ppm/syrup** | **DF 1ppm/syrup** | **F-0.565ppm/polle** | **DF 10ppm/syrup** | **DF 5.65 ppm/polle** | **CR-1 0ppm/syrup** | **CR-2 0ppm/syrup** | **DF 2ppm/syrup** | **FT 10ppm/syrup** | **CR-1 0ppm/syrup** | **CR-2 0ppm/syrup** | **DF 0.2ppm/syrup** | **CN 0.08ppm/syrup** | **FT 1ppm/syrup** | **MT 1ppm/syrup** |
| **28-Jun** |  |  |  |  |  | 19.03 | 14.49 | 15.86 | 17.95 |  |  |  |  |  |  |
| **8-Jul** |  |  |  |  |  | 25.65 | 22.15 | 19.78 | 22.58 |  |  |  |  |  |  |
| **9-Jul** | 7.38 | 8.80 | 10.68 | 10.75 | 3.45 |  |  |  |  |  |  |  |  |  |  |
| **15-Jul** |  |  |  |  |  | 21.94 | 21.07 | 14.61 | 22.06 |  |  |  |  |  |  |
| **16-Jul** | 14.85 | 12.64 | 18.59 | 3.59 | 0.89 |  |  |  |  |  |  |  |  |  |  |
| **21-Jul** |  |  |  |  |  | 21.53 | 23.06 | 16.91 | 22.56 |  |  |  |  |  |  |
| **22-Jul** | 14.59 | 16.75 | 17.45 | 5.88 | 1.62 | 21.12 | 20.76 | 6.11 | 16.76 |  |  |  |  |  |  |
| **27-Jul** |  |  |  |  |  | 14.51 | 13.24 | 1.03 | 11.57 |  |  |  |  |  |  |
| **28-Jul** |  |  |  |  |  | 13.79 | 13.15 | 1.90 | 9.52 |  |  |  |  |  |  |
| **29-Jul** | 21.27 | 17.82 | 20.46 | 14.38 | 0.12 |  |  |  |  |  |  |  |  |  |  |
| **3-Aug** |  |  |  |  |  | 15.41 | 13.52 | 5.15 | 12.31 |  |  |  |  |  |  |
| **4-Aug** |  |  |  |  |  | 14.88 | 13.70 | 1.22 | 11.96 |  |  |  |  |  |  |
| **6-Aug** | 21.89 | 19.66 | 20.21 | 19.78 | 3.02 |  |  |  |  |  |  |  |  |  |  |
| **8-Aug** |  |  |  |  |  | 16.23 | 14.86 | 0.05 | 12.62 |  |  |  |  |  |  |
| **12-Aug** | 22.26 | 23.55 | 20.77 | 24.85 |  |  |  |  |  |  |  |  |  |  |  |
| **13-Aug** |  |  |  |  |  |  |  |  |  | 23.01 | 16.09 | 14.97 | 14.18 | 18.28 | 15.08 |
| **16-Aug** |  |  |  |  |  | 18.39 | 15.60 | 0.00 | 18.86 |  |  |  |  |  |  |
| **18-Aug** | 17.63 | 18.38 | 18.72 | 13.50 |  |  |  |  |  |  |  |  |  |  |  |
| **24-Aug** |  |  |  |  |  |  |  |  |  | 21.26 | 16.32 | 20.76 | 17.43 | 23.08 | 18.65 |
| **25-Aug** |  |  |  |  |  | 19.88 | 18.37 |  | 22.64 | 25.14 | 21.56 | 27.74 | 25.17 | 28.81 | 26.84 |
| **26-Aug** | 15.17 | 11.42 | 18.63 | 11.99 |  |  |  |  |  | 27.73 | 26.86 | 30.73 | 28.67 | 22.27 | 29.32 |
| **1-Sep** |  |  |  |  |  |  |  |  |  | 23.77 | 19.22 | 25.07 | 23.95 | 27.90 | 26.11 |
| **5-Sep** |  |  |  |  |  |  |  |  |  | 25.14 | 21.56 | 27.74 | 25.17 | 28.81 | 26.84 |
| **6-Sep** |  |  |  |  |  | 23.00 | 21.68 |  | 31.86 |  |  |  |  |  |  |
| **10-Sep** | 18.82 | 9.73 | 26.34 | 22.46 |  |  |  |  |  |  |  |  |  |  |  |
| **15-Sep** |  |  |  |  |  | 21.31 | 20.47 |  | 38.45 |  |  |  |  |  |  |
| **17-Sep** | 15.75 | 14.26 | 30.04 | 26.76 |  |  |  |  |  |  |  |  |  |  |  |
| **21-Sep** |  |  |  |  |  | 21.00 | 18.04 |  | 36.60 | 27.08 | 29.13 | 34.44 | 28.20 | 24.86 | 29.32 |
| **24-Sep** | 16.44 | 17.39 | 29.47 | 17.90 |  |  |  |  |  |  |  |  |  |  |  |
| **27-Sep** |  |  |  |  |  |  |  |  |  | 13.68 | 29.88 | 33.45 | 30.74 | 16.68 | 22.93 |
| **29-Sep** | 16.37 | 13.06 | 29.54 | 19.94 |  |  |  |  |  |  |  |  |  |  |  |
| **4-Oct** |  |  |  |  |  |  |  |  |  | 14.39 | 16.20 | 41.41 | 19.35 | 17.33 | 14.47 |
| **5-Oct** |  |  |  |  |  |  |  |  |  |  |  |  |  |  |  |
| **7-Oct** | 20.65 | 5.51 | 28.18 | 20.19 |  |  |  |  |  |  |  |  |  |  |  |
| **13-Oct** |  |  |  |  |  |  |  |  |  | 20.18 | 20.63 | 31.82 | 27.41 | 22.14 | 18.90 |
| **19-Oct** |  |  |  |  |  | 19.59 | 19.03 |  | 20.95 |  |  |  |  |  |  |
| **21-Oct** | 23.79 | 1.53 | 22.40 | 28.40 |  |  |  |  |  |  |  |  |  |  |  |
| **27-Oct** |  |  |  |  |  |  |  |  |  | 31.34 | 29.89 | 41.98 | 40.19 | 33.36 | 30.19 |
| **30-Oct** | 26.73 |  | 25.30 | 35.46 |  |  |  |  |  |  |  |  |  |  |  |
| **4-Nov** | 27.44 |  | 21.99 | 37.66 |  |  |  |  |  |  |  |  |  |  |  |
| **15-Nov** |  |  |  |  |  |  |  |  |  | 48.37 | 48.19 | 60.17 | 56.81 | 51.13 | 47.95 |
| **18-Nov** | 33.75 |  | 22.25 | 47.23 |  |  |  |  |  |  |  |  |  |  |  |
| **25-Nov** |  |  |  |  |  | 47.53 | 37.74 |  | 45.86 |  |  |  |  |  |  |
| **26-Nov** | 39.66 |  | 21.91 | 50.39 |  |  |  |  |  |  |  |  |  |  |  |
| **1-Dec** |  |  |  |  |  |  |  |  |  | 62.34 | 63.18 | 74.23 | 70.46 | 66.43 | 62.35 |
| **3-Dec** | 43.64 |  | 21.41 | 29.30 |  |  |  |  |  |  |  |  |  |  |  |
| **13-Dec** |  |  |  |  |  | 64.81 | 51.75 |  | 62.00 |  |  |  |  |  |  |
| **17-Dec** | 50.76 |  | 28.47 | 0.71 |  |  |  |  |  |  |  |  |  |  |  |
| **5-Jan** |  |  |  |  |  |  |  |  |  | 92.61 | 95.60 | 0.00 | 0.00 | 100.39 | 92.97 |
| **1-Feb** |  |  |  |  |  | 108.91 | 93.76 |  | 108.11 |  |  |  |  |  |  |
| **7-Feb** |  |  |  |  |  |  |  |  |  | 3.81 | 127.15 |  |  | 124.23 | 0.00 |
| **16-Feb** | 107.14 |  | 14.29 |  |  |  |  |  |  |  |  |  |  |  |  |
| **28-Feb** |  |  |  |  |  |  |  |  |  | 0.00 | 147.03 |  |  | 0.00 |  |
| **1-Mar** |  |  |  |  |  | 133.75 | 118.44 |  | 136.85 |  |  |  |  |  |  |
| **9-Mar** |  |  |  |  |  | 141.82 | 124.67 |  | 145.90 |  |  |  |  |  |  |
| **17-Mar** |  |  |  |  |  | 148.28 | 128.57 |  | 152.54 |  |  |  |  |  |  |
| **23-Mar** |  |  |  |  |  | 154.08 | 131.34 |  | 157.36 |  |  |  |  |  |  |
| **29-Mar** |  |  |  |  |  | 159.04 | 133.66 |  | 161.37 |  |  |  |  |  |  |
| **2-Apr** | 133.10 |  |  |  |  |  |  |  |  |  |  |  |  |  |  |
| **7-Apr** |  |  |  |  |  | 166.80 | 136.13 |  | 166.52 |  |  |  |  |  |  |
| **13-Apr** |  |  |  |  |  | 172.59 | 138.63 |  | 172.62 |  |  |  |  |  |  |
| **19-Apr** |  |  |  |  |  | 177.64 | 141.88 |  | 175.61 |  |  |  |  |  |  |
| **26-Apr** |  |  |  |  |  | 181.88 | 38.93 |  | 37.89 |  |  |  |  |  |  |
| **3-May** |  |  |  |  |  | 185.32 | 30.47 |  | 33.22 |  |  |  |  |  |  |
| **10-May** |  |  |  |  |  | 34.93 | 28.37 |  | 20.60 |  |  |  |  |  |  |
| **17-May** |  |  |  |  |  | 26.01 | 28.76 |  | 14.91 |  |  |  |  |  |  |
| **26-May** |  |  |  |  |  | 22.57 | 17.50 |  | 12.86 |  |  |  |  |  |  |
| **2-Jun** |  |  |  |  |  | 16.76 | 27.14 |  | 9.89 |  |  |  |  |  |  |
| **14-Jun** |  |  |  |  |  | 11.18 | 26.34 |  | 10.49 |  |  |  |  |  |  |
| **21-Jun** |  |  |  |  |  | 14.36 | 27.64 |  | 11.92 |  |  |  |  |  |  |
| **28-Jun** |  |  |  |  |  | 12.24 | 31.03 |  | 12.21 |  |  |  |  |  |  |
| **8-Jul** |  |  |  |  |  | 13.31 | 21.93 |  | 14.87 |  |  |  |  |  |  |
| **26-Jul** |  |  |  |  |  |  |  |  |  |  |  |  |  |  |  |
