# Supplementary material for: Comparison of long-term changes in size and longevity of bee colonies in mid-west Japan and Maui with and without exposure to pesticide, cold winters, and mites
Source: PeerJ. 2020 Jul 28;8:e9505. doi: 10.7717/peerj.9505 (PMC7394064; doi:10.7717/peerj.9505)

Comparison of long-term changes in size and longevity of bee colonies in mid-west Japan and Maui with and without exposure to pesticide, cold winters, and mites

[Short Title]
Bee longevity comparison in two regions

Toshiro Yamada[1], Kazuko Yamada[2]
[1] Graduate School of Natural Science & Technology, Kanazawa University, Kanazawa, Ishikawa, Japan
[2] Freelance, Kanazawa, Ishikawa, Japan

Corresponding Author:
Toshiro Yamada[1]
Email address: yamatoshikazu0501@yahoo.co.jp

Supplementary Data

Table S1. Experimental conditions of five long-term field experiments.

Experiment name	2010 experiment	2011/2012 experiment	2012/2013 experiment	2013/2014 experiment	2014/2015 experiment (This work)
Experimental period	July 18 to November 21, 2010 (126 days)	From July 9, 2011 to April 2, 2012 (268 days)	From June 28, 2012 to July 26, 2013 (393 days)	From August 13, 2013 to February 28, 2014 (199 days)	From October 22, 2014 to July 20, 2015 (271 days)
Experimental site (latitude and longitude)	Shika (mid-west Japan) (37º1′9″ N, 165º46′14″ E)	Same as left	Same as left	Same as left	Maui (Hawaii) (20º55'00.2" N, 156º30'39.2" W)
Object of Study	To investigate the possibility for a neonicotinoid of causing a CCD	To investigate the difference in the long-term influence on a bee colony between toxic sugar syrup (honey) as an energy source and toxic pollen pase (bee bread) as a protein source which are exposed to a neonicotinoid	To investigate the difference in the long-term influence on a bee colony between a neonicotinoid and a organophosphate which are administered through sugar syrup	To investigate the difference in the long-term influence on a bee colony between a neonicotinoid and a organophosphate which are administered through sugar syrup under lower concentrations than in the previous work	To compare the long-term changes in colony size and apparent longevity between Shika (mid-west Japan) with mites and cold winter and Maui (Hawaii) without both
Circumstances around Experimental Site					
**Limitation of honeybee activities**	Honeybees can freely forage about for food in a hive or in fields	Same as left	Same as left	Same as left	Same as left
**Impact of other pesticide than the administered one on the environment**	A pesticide-free watering place & a pesticide-free field of flowers in the apiary	Same as left	Same as left	Same as left	Experimental site is located in organically-grown macadamia trees
**Aerial-crop-dusting farmland near exptl. Site**	Nothing	Nothing	Nothing	Nothing	Nothing
**Seasonal changes**	Distinct	Distinct	Distinct	Distinct	Indistinct (without cold winter)
Experimental Conditions					
**Initial numbers of apiaries, colonies and combs (frames)**	1 apiary (private), 8 colonies per apiary, 6 combs per c	1 apiary (private), 5 colonies per apiary, 3 combs per c	1 apiary (private), 4 colonies per apiary, 3 combs per c	1 apiary (private), 6 colonies per apiary, 3 combs per c	1 apiary (private), 6 colonies per apiary, 3 combs per colony
**Initial number of bees per colony**	9000 to 1300 bees (accurately counted on photos)	1700 to 3400 bees (accurately counted from photos)	5600 to 7100 bees (accurately counted on photos)	5400 to 7600 bees (accurately counted on photos)	5400 to 7600 bees (accurately counted on photos)
**Initial number of capped brood per colony**	1.5 to 7 full-surfaces on comb converted from the sum total of every area occupied by capped (obtained from photos)	2600 to 6100 capped brood (accurately counted from photos)	4000 to 5700 capped brood (accurately counted from photos)	4200 to 7600 capped brood (accurately counted from photos)	4200 to 7600 capped brood (accurately counted from photos)
**Kind of pesticide**	Dinotefuran, Clothianidin	Dinotefuran	Dinotefuran, Fenitrothion	Dinotefuran, Clothianidin, fenitrothion, Malathion	Dinotefuran, Clothianidin, fenitrothion
**Concentration of pesticide**	Dinotefuran: 1, 2, 10 ppm, Clothianidin: 0.4, 2, 4 ppm	Dinotefuran: 1 & 10 ppm in sugar syrup, 0.565 & 5.65 in pollen paste	Dinotefuran: 2 ppm in sugar syrup, Fenitrothion: 10 ppm in sugar syrup	Dinotefuran: 0.2 ppm in sugar syrup, Clothianidin: 0.08 ppm in sugar syrup, Fenitrothion & Malathion: 1 ppm in sugar syrup	Dinotefuran: 0.2 ppm in sugar syrup, Clothianidin: 0.08 ppm in sugar syrup, Fenitrothion 1 ppm in sugar syrup (Same as 2013/2014 experiment in Shika)
**Origin of a queen**	Unknown in detail (*Apis mellifera*) Bee colonies purchased from a bee farm	Same as left	Same as left	Same as left	Sister relationship among queens
Experimental Methods					
**Interval of experiment**	About one-week interval	About one-week interval	About one-week or two-weeks interval	Same as left	About two-weeks or more interval
**Administration period of pesticide**	Till colony extinction or from July 18, 2010 to November 21 (126 days)	Till colony extinction or from July 9, 2011 to December 3 (147 days)	Till colony extinction or from July 21, 2012 to August 16 (26 days)	Till colony extinction or from September 5, 2013 to December 1 (87 days)	Till colony extinction or from October 23, 2014 to April 4, 2015 (163 days)
**Starting time of each observation**	Just after dawn if possible (before bees go out to forage)	Same as left	Same as left	Same as left	Same as left
**Vehicle to administer a pesticide**	Both sugar syrup and pollen paste	Either sugar Syrup or pollen paste	Sugar syrup	Sugar syrup	Sugar syrup
**Administration method of pesticide**	A pesticide was dissolved in sugar syrup and pollen was kneaded with toxic sugar syrup containing the pesticide. Both toxic sugar syrup and toxic pollen paste were fed into a hive.	A pesticide was dissolved in sugar syrup or pollen was kneaded with toxic sugar syrup containing the pesticide. Either toxic sugar syrup or toxic pollen paste was fed into a hive.	A pesticide was dissolved in sugar syrup with toxic sugar syrup containing the pesticide. Only toxic sugar syrup was fed into a hive.	A pesticide was dissolved in sugar syrup with toxic sugar syrup containing the pesticide. Only toxic sugar syrup was continuously fed into a hive with an auto-feeding system composed of 10 L (14 kg syrup) container	Same as left
**Counting method of the number of adult bees**	Roughly counted from photos of combs with bees and bees left in a hive after every comb was removed from it	Directly counted with accuracy from photos of combs with bees and bees left in a hive after every comb was removed from it with the help of a automatic counting software	Same as left	Same as left	Same as left
**Counting method of the number of capped brood**	Expressed by the number of surfaces on combs (frames) which are entirely occupied by capped brood using the photos of combs	Acurately counted from photos of combs without bees after shaking the bees off each comb	Same as left	Same as left	Same as left
**Total intake of pesticide per colony**	Calculated from the sugar syrup & pollen paste with pesticide consumed by honeybees	Calculated from the sugar syrup or pollen paste with pesticide consumed by honeybees	Calculated from the sugar syrup with pesticide consumed by honeybees	Same as left	Same as left
**Estimation of the intake of pesticide per bee**	Unestimated	Estimated from dividing the total intake of pesticide per colony by the total number of initial & newly-emerged honeybees	Same as left	Same as left	Same as left
**Counting method of number of dead bees**	Dead bees were accurately counted one by one inside and outside a hive which was placed on a large tray	Same as left	Same as left	Same as left	Same as left
**Confirmation and record methods of a queen**	A photographic record of the exsistence of a queen in each colony	Same as left	Same as left	Same as left	Same as left
Publications of research results					
**Experimental Results**	T. Yamada et at. (2012). *Jpn. J. Clin. Ecol.* 21(1): 10-23.	T. Yamada et al. (2018a). *J. Biol. Ser.* 1(3): 084-107.	Yamada et al. (2018b). *J. Biol. Ser.* 1(3): 108-137	T. Yamada et al. (2018d). *J. Biol. Ser.* 1(4): 187-207.	T. Yamada et al. (2018c). *J. Biol. Ser*. 1(4): 156-186.
**Apparent longevity**	Y. Yamada et al. (2019). A mathematical model for estimation of long-term change in apparent longevity of honeybee colony. *Scientific Reports* 9: 4102.				**Submitted to Peer J (this work)**
	T. Yamada, et at. (unpublished). Seasonal change in apparent longevity of bee colony exposed to pesticide in a field experiment. Under submission elsewhere.				

Table S2. Field experimental procedures.

It is generally difficult to replicate a field experiment including a variety of extraneous disturbance factors.

Someone may enable to replicate and our field experiment and check the outcomes, minimizing the uncertainties in the conduction of experiment according to the following procedures.

1) Select an experimental site where there are no crop-dusting area nearby and no animals to cause a great damage to a honeybee colony such as a bear to reduce uncontrollable disturbances.

2) Reduce or compensate the effect of the hive arrangement, for example, by placing control colonies at both sides of experimental colonies.

3) Start a field experiment after the initial size of each colony such as the numbers of adult bees and capped brood becomes almost the same.

4) Conduct a field experiment on a fine or cloudy day because bad weather makes honeybees nervous.

5) Avoid swarming season of honeybees because swarming disturbs the unity of experimental conditions among bee colonies.

6) Begin conducting an experiment just after dawn before foraging bees go out anywhere.

7) Conduct an experiment every two weeks or so if possible because capping period of brood is 12 days.

8) Leave observation results on photographic record as much as possible because the results can be checked again afterwards.

9) Attach a name plate by which prime conditions are described at the front of each hive.

10) Number both sides of each comb and to set each comb at a fixed position to keep the experimental conditions constant.

11) Take a photograph of the whole view of the experimental site.

12) Take a photograph of the front of a hive.

13) Take photographs of both sides of a comb with honeybees while pulling out a comb in comb-number order and afterwards to put in another empty hive which is prepared in advance.

14) Take an enlarged photograph of a queen bee when the queen is found on a comb or inside a hive.

15) Take a photographs the inside of the hive in which honeybees are left (four walls and the bottom) after pulling out every comb from the hive.

16) Take a photograph of the comb without honeybees after shaking them off the comb in comb-number order and to put every comb back where it belonged.

17) Measure the amount of food (sugar syrup, pollen paste) consumed by honeybees by a balance accurate to about 0.1 g.

18) Feed a fixed weight of a new food (sugar syrup only in Maui) in the hive after remove the old food.

19) Take a photograph of the irregularity such as queen cells, hive-beetles, mites (no exists in Maui), wax worm larvae, attacks of Japanese giant hornet (no exists in Maui) and so on during experimenting while recording the results on a datasheet.

20) Record the observational results in the research note after discussing them with experimenters.

21) Add a caption of a photograph of each side of every comb and each wall of the hive-inside to understand the condition on a computer, for example, "20150130_DF3-1B" denotes a photograph of the back side of the first comb of DF-3 (the third colony of dinotefuran-exposed colonies) which was taken on January 30, 2015.

Figure S1. Seasonal change apparent longevity of control colony and pesticide-exposed colony in Shika (mid-west Japan).

CR-1, CR-2: Control (pesticide-free) colony, DF: Dinotefuran-exposed colony, CN: Clothianidin-exposed colony, FT: Fenitrothion-exposed colony, MT: Malathion-exposed colony, DF & CN are neonicotinoids, FT & MT are organophosphates. Using the numbers of adult bees and capped brood obtained from 2011/2012, 2012/2013 and 2013/2014 field experiments, apparent longevity in each colony was estimated from a mathematical model proposed by Y. Yamada et al. (2019). A pesticide (DF, CN, FT, MT) is administered to each colony through sugar syrup. Details of the long-term field experiments are described in previous papers (T. Yamada et al., 2018a, 2018b, 2018d). The apparent longevity begin to increase from the end of September with the approach of winter and it drops rapidly just after it reaches its maximum at the end of overwintering. Strong colonies succeed in overwintering through such a course, but weak colonies become extinct during overwintering. Until extinct, the apparent longevity of pesticide-exposed colony (DF, CN, FT, MT) changes in the same way as that of control colony.

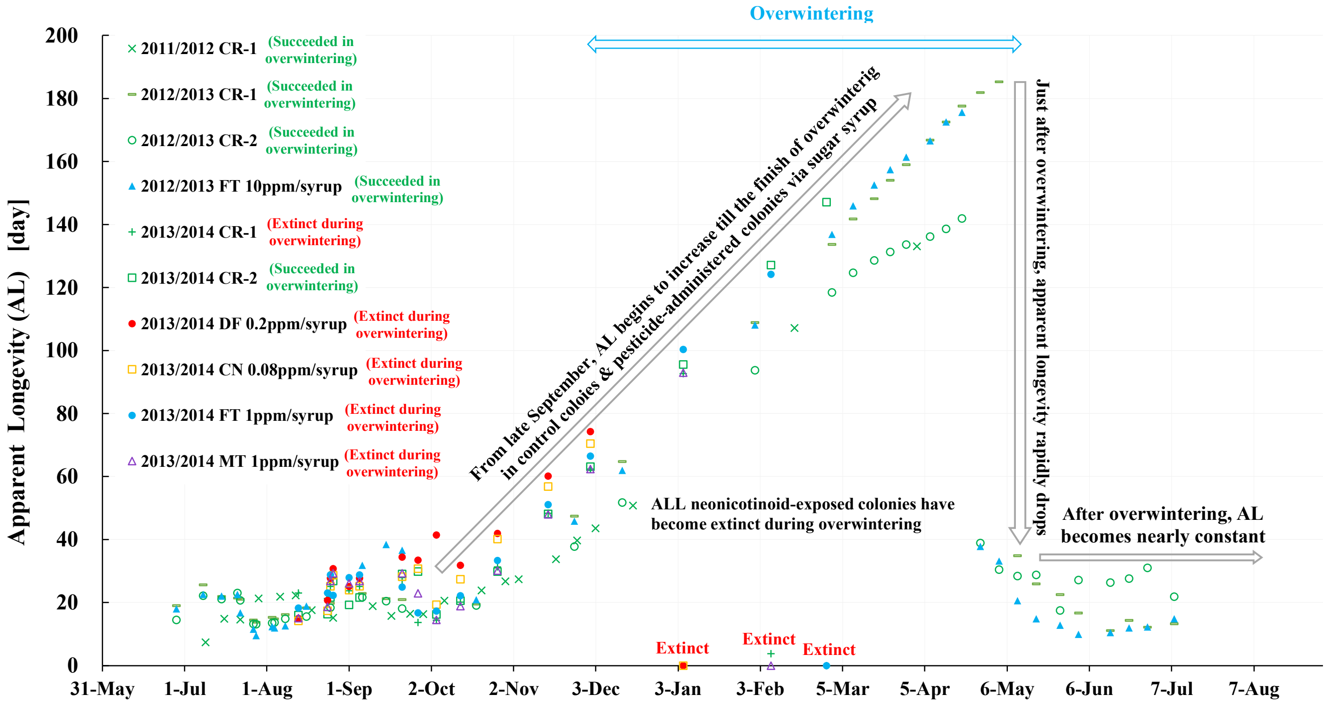

Figure S2. Atmospheric temperature (a) and monthly precipitation (b) in Maui and Shika (mid-west Japan). Tmax= Daily maximum temperature; Tmin= Daily minimum temperature; Rainfall= Monthly precipitation. Data are available at https://shortvacation.jp/beach/hawaii/maui/. (Maui) and http://www.data.jma.go.jp/obd/stats/etrn/view/nml_amd_ym.php?prec_no=56&block_no=0564&year=2014&month=06&day=&view=p1. (Shika, mid-west Japan)

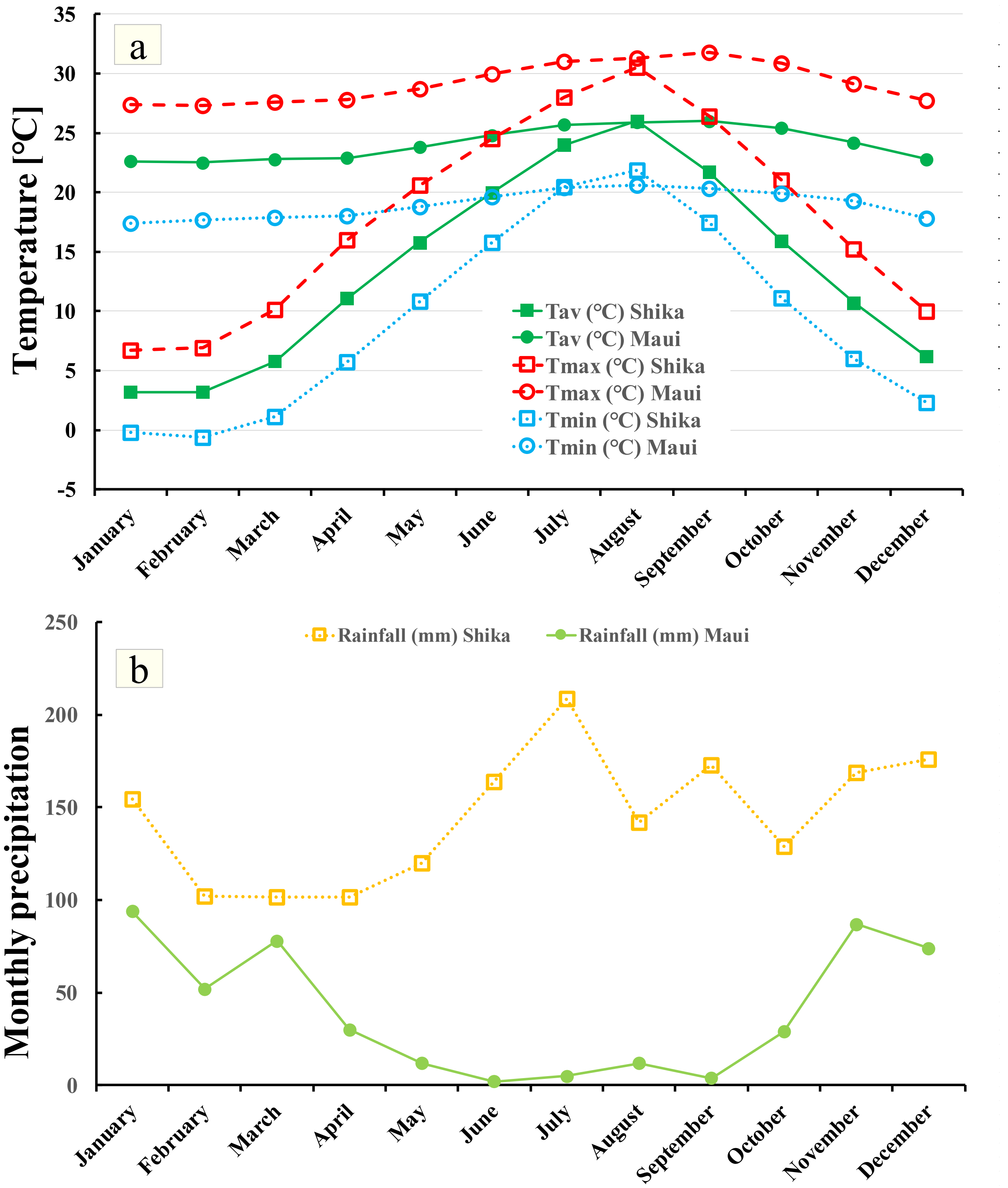

Supplement: Supplemental Information 8 [file peerj-08-9505-s008.pdf]
